# An automated and parallelised DIY-dosing unit for individual and complex feeding profiles: Construction, validation and applications

**DOI:** 10.1371/journal.pone.0217268

**Published:** 2019-06-19

**Authors:** Sabine G. Wagner, Christoph Mähler, Ingmar Polte, Jeremy von Poschinger, Hannes Löwe, Andreas Kremling, Katharina Pflüger-Grau

**Affiliations:** 1 TU Munich, Systems Biotechnology, Faculty of Mechanical Engineering, Garching, Germany; 2 TU Munich, Biochemical Engineering, Faculty of Mechanical Engineering, Garching, Germany; Universitatsstr Beyreuth, GERMANY

## Abstract

Since biotechnological research becomes more and more important for industrial applications, there is an increasing need for scalable and controllable laboratory procedures. A widely used approach in biotechnological research to improve the performance of a process is to vary the growth rates in order to find the right balance between growth and the production. This can be achieved by the application of a suitable feeding strategy. During this initial bioprocess development, it is beneficial to have at hand cheap and easy setups that work in parallel (e.g. in shaking flasks). Unfortunately, there is a gap between these easy setups and defined and controllable processes, which are necessary for up-scaling to an industrial relevant volume. One prerequisite to test and evaluate different process strategies apart from batch-mode is the availability of pump systems that allow for defined feeding profiles in shaking flasks. To our knowledge, there is no suitable dosing device on the market which fulfils the requirements of being cheap, precise, programmable, and parallelizable. Commercially available dosing units are either already integrated in bioreactors and therefore inflexible, or not programmable, or expensive, or a combination of those. Here, we present a LEGO-MINDSTORMS-based syringe pump, which has the potential of being widely used in daily laboratory routine due to its low price, programmability, and parallelisability. The acquisition costs do not exceed 350 € for up to four dosing units, that are independently controllable with one EV3 block. The system covers flow rates ranging from 0.7 μL min^-1^ up to 210 mL min^-1^ with a reliable flux. One dosing unit can convey at maximum a volume of 20 mL (using all 4 units even up to 80 mL in total) over the whole process time. The design of the dosing unit enables the user to perform experiments with up to four different growth rates in parallel (each measured in triplicates) per EV3-block used. We estimate, that the LEGO-MINDSTORMS-based dosing unit with 12 syringes in parallel is reducing the costs up to 50-fold compared to a trivial version of a commercial pump system (~1500 €) which fits the same requirements. Using the pump, we set the growth rates of a *E*. *coli* HMS174/DE3 culture to values between 0.1 and 0.4 h^-1^ with a standard deviation of at best 0.35% and an average discrepancy of 13.2%. Additionally, we determined the energy demand of a culture for the maintenance of the pTRA-51hd plasmid by quantifying the changes in biomass yield with different growth rates set. Around 25% of total substrate taken up is used for plasmid maintenance. To present possible applications and show the flexibility of the system, we applied a constant feed to perform microencapsulation of *Pseudomonas putida* and an individual dosing profile for the purification of a his-tagged eGFP via IMAC. This smart and versatile dosing unit, which is ready-to-use without any prior knowledge in electronics and control, is affordable for everyone and due to its flexibility and broad application range a valuable addition to the laboratory routine.

## Introduction

Controllable pump systems are necessary for a broad range of applications including the process control of biotechnological cultures. This is at the same time a very challenging way to perform dosing as often time-depending and non-linear profiles are required [1] In industrial applications usually the fed-batch-mode is preferred over a mere batch process [2–4]. A constant but limiting substrate feed reduces the probability of overflow metabolism as potential and unwanted side-product formation [5], and prevents substrate inhibition, which in turn lead to higher cell densities [6] and consequently to enhanced biomass production [7, 8].

The basic process development however, often takes place in shaking flasks [9], because of their unbeaten capacity to be operated in parallel and their fast handling. The great disadvantage of these or other parallel cultivation systems as the miniaturized bioREACTOR48, is the difficulty to set the growth rate of the cells by providing a controlled and continuous feed. Nevertheless, especially during the production of heterologous proteins, a centrepiece of bioprocess engineering is to balance the growth and heterologous production. As the productivity of a cell is tightly linked to the availability of cellular resources like ribosomes or RNA-polymerases, which themselves are coupled to growth [10], it is possible to further optimise the productivity of a heterotrophic process by choosing the best dosing-profile for the nutrient supply [11,12]. A feeding strategy can be used to set the growth rate of bacterial cells and consequently the production performance of the culture. Other approaches to set the growth rate in bacterial cultures are the reduction of the incubation temperature or by using different carbon sources [13–16]. A lower temperature than the optimum during growth can even have positive effects on protein folding [17], however, it can also lead to stress-alarmed cells and the reduction of biocatalytic reaction velocities [18]. The use of various carbon sources for the adaption of growth rates also has to be carefully examined. The metabolization of different carbon sources is often performed following distinct metabolic routes and therefore results in cells having altered metabolic regimes [19] that do not allow comparability of the cultures. Thus, we argue that the best way to implement different growth rates in shaking flasks is to define the availability of the substrate. Apart from feeding, there are other possibilities to achieve this, e.g. the use of compartments, which release nutrients over time [3,20], the enzymatic splitting of non-consumable polysaccharides as e.g. in the EnBase technology [21,22], or targeting the uptake system or the metabolic pathway via genetic modifications [14,23]. These are proportionately cheap methods but they can be either difficult to control, or they are linear instead of exponential, or they can cause troubles during the scale-up.

An easy and straightforward solution is the use of well-established and commercially available pump systems [2] to supply a constant feed of nutrients to a bacterial culture. These pumps are mostly able to dose with a high accuracy and can provide small and time-dependent flow rates. There are a couple of suitable pump systems on the market that can be used with shaking flasks and connected to a process-control-software, for instance in the LAMBDA pump portfolio (https://www.lambda-instruments.com/). The LAMBDA PRECIFLOW peristaltic pump for example, fits the requirements for substrate feeding in shaking flasks. At a list price of 1450 € the pump can convey flow rates between 0.01 and 60 mL h^-1^. Another option is the use of syringe pumps with finite dosing volumes, as during a feeding-strategy for shaking flasks only small volumes are dosed. Moreover, the use of syringe pumps can be an advantage, because hoses of peristaltic become stretched over time and the pumps need to be calibrated in periodic intervals. The syringe pump LAMBDA VIT-FIT (1718 €) for example, which is limited in push travel and therewith in total volume, can work in a ratio between 0.08 and 80 mm min^-1^ (the volumetric flow rate depends on syringe type) dosing. However, both systems can only provide a single flow rate out of one reservoir. The parallel operating pumps in the same price segment, e.g. in the ISMATEC pump collection, either lack programmable dosing profiles or the parallelizability is limited not least because of the costs. A dosing system which fulfils all requirements, e.g. the pump ISM 915, can cost 3500 € and more. Considering the accuracy at very low flow rates dosed by commercially available pumps, the dosing units for medical applications are known as the bests. For example, the commercially available diaphragm pump Type 7615 (Bürkert), which is approved for medical applications, doses 5 μl per stroke with an accuracy of +/-2%. This pump system is even in the same range of price as the here presented syringe pump but neither parallelized nor programmable. A totally different and smart solution for dosing via the headspace of shaking flasks is provided by the company Aquila Biolabs with the LIS (Liquid Injection System). Although the LIS is programmable, it must be noted that the LIS generates operating costs and the feed is provided in droplets which narrows the lower limit of flow rate.

To find the right pump system for the desired experimental design is not easy and means to compromise between costs, features and handling. An ideal dosing system for parallel experiments in shaking flasks or comparable cultivation systems should not only be cheap, parallelisable, controllable, and continuous, but also flexible and simple to operate.

For that reason, the aim of this work was to design and construct an automated dosing unit with low acquisition costs, easy applicability, and a maximum of flexibility concerning the feeding profile. Here, we present the design and validation of a LEGO-MINDSTORMS-based syringe pump, as an easy and cheap do-it-yourself (DIY) version of an automated and parallel feeding system to control growth. In contrast to many other open-source electronic prototyping platforms as for example Arduino, the concept of LEGO-constructions is not based on any deeper prior knowledge [24,25] and therefore open for a broad community. In this work, the third-generation of the LEGO-MINDSTORMS-series EV3 is employed, which does not only provide several interfaces for communication, but is also accessible for different programming languages. Since LEGO-constructions are very popular, several online marketplaces, like BrickOwl, are providing an easy access to single bricks. The main costs of the dosing unit presented here can basically be reduced to the LEGO-MINDSTORMS-EV3 brick (~ 195 €) and the large servo motor (~27 €).

The LEGO-based dosing device designed in this work, was tested in several applications to show its broad applicability and flexibility. Apart from using it as a feeding system for parallel bacterial cultures in shaking flasks, we also produced uniform spherical micro-particles, and performed complex flux profiles for protein purification. In addition to these biotechnological applications, the tool might also be useful to enhance complex drug delivery assays or other microfluidic applications in emerging fields such as regenerative medicine and tissue engineering [26]. Therefore, this system has the potential to become a widely used tool for individual dosing profiles, which can be applied for a broad range of miniaturised but scalable research scopes.

## Results and discussion

### Components of the automated dosing unit

**Fig 1:**
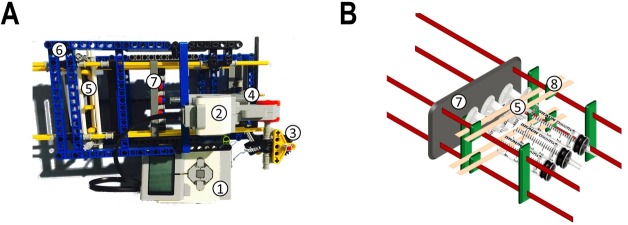
**The LEGO-dosing unit (A) and** the **syringe clamping device of the prototype (B).** The dosing unit, which is controlled by the programmable MINDSTORMS-EV3 brick (A.1), consists of a EV3 large servo motor (A.2), gear-down modules (the prototype version contains two gearboxes) (A.3), a linear actuator (A.4), the syringe clamping device (A.5) and the housing (A.6) which is stabilising the construct. The syringe clamping device enables the use of different syringe types. The contact device (A and B.7) transfers the rotation initiated by the EV3 brick into piston movement. The syringes can be fixated by the locator (B.5). Three 10 mL syringes inserted into the movable brackets (B.8) are displayed exemplary.

The programmable LEGO-MINDSTORMS-EV3 brick (Fig 1A.1) was chosen as the centrepiece of the dosing unit. This EV3 brick is powered by a TI Sitara 300 MHz ARM9 core processor and is running under a Linux-based operating system. It is equipped with an on-board program storage including 16 MB of Flash memory, 64 MB of RAM, and a Mini SDHC card reader for up to 32 GB of expanded memory. The EV3 brick serves as both, control center and power station of the pump. To create simple dosing profiles, like for example linear feeding, the EV3 Programmer App, provided at the official LEGO-webstore (https://www.lego.com/en-gb/MINDSTORMS/apps/ev3-programmer-app?ignorereferer=true) can be used. For more complex profiles however, a third-party software is necessary. We chose the commonly used MATLAB software to create the profiles used in this work (scripts are deposited in S1 and S6 Files). The EV3 brick has to be connected to the device on which MATLAB is operating, either by Bluetooth, WLAN, or by USB. Because the cultivation of bacterial cells routinely takes place inside enclosed incubators to provide the appropriate temperature, the favoured type of communication is wireless. However, in this setup, battery life is only around eight hours, thus for long-term dosing-profiles a USB connection is recommended, which decouples the process from the battery charge. After the communication between the building block and the software is established, several commands can be executed. Up to four pump units can be regulated separately per EV3 block (Fig 1A.1). Each unit consists of the EV3 large servo motor (Fig 1A.2), the optional gear-down modules (Fig 1A.3), a linear actuator (Fig 1A.4), the syringe-clamping-device (Fig 1A.5), and the housing to stabilise the construction (Fig 1A.6). Once it is built, the servo motors of the pump units can be connected to the EV3 control block, using one out of the four RJ12 jacks. An order list can be found in S1 Table.

Prior to starting the dosing process, the syringes have to be filled and mounted in the syringe clamping device. This is facilitated by retracting completely the piston rod of the linear actuator. Next, up to 3 syringes are inserted into the adjustable brackets (Fig 1B.8) and fixed mechanically by moving down the locator (Fig 1B.5). The insertion of 1 mL, 20 mL, and 50 mL syringes is possible, however for 50 mL syringes the locator has to be removed. To avoid so-called trumpet curves caused by inaccuracies in the feeding profile, we recommend to manually move the piston until a first droplet can be observed and to assure that the contact device (Fig 1B.7) is tightly in touch. By this, the automated dosing device is successfully mounted and prepared for starting the dosing process.

### Technical characteristics

**Fig 2:**
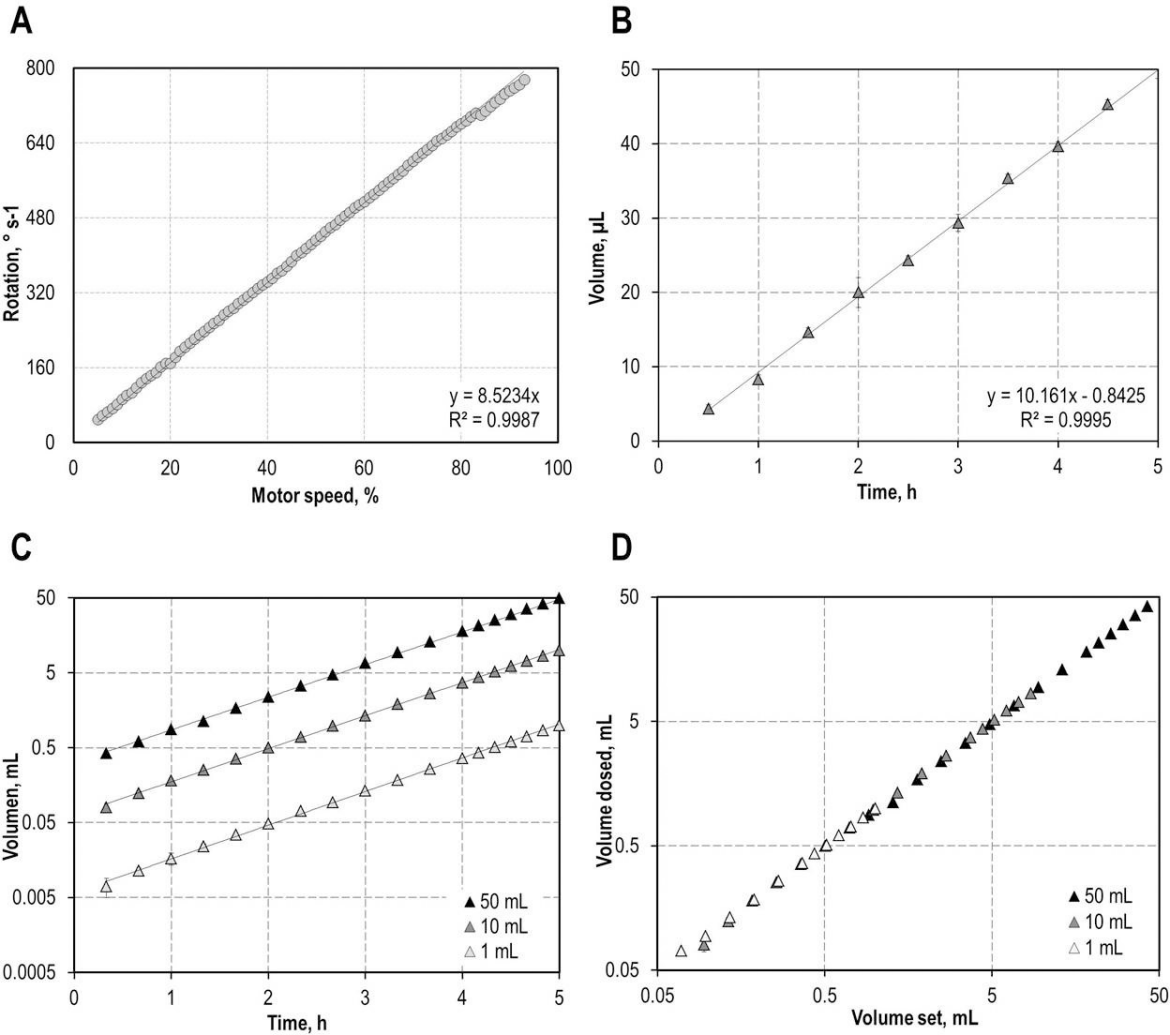
Different setups were tested to validate the reliability of the dosing unit. In (A) the motor speed was correlated to the rotations performed. Figure (B) shows the volume conveyed over time for 10 mL syringes at a flow rate of 10 μL h^-1^. (C) The complex feed function *V*_*F*_(*t*) = *b*∙*e*^*a*∙*t*^ was tested with 1, 10, and 50 mL-syringes. The volume dosed was monitored gravimetrically over time. (D) Correlation of the actual volume dosed with the volume set.

Although the motor with a running torque of 20 N cm is using a tacho-feedback for precise control within one degree of accuracy, we recommend the use of the built-in rotation sensor to define and control the volume conveyed by the pump to avoid delays and to compensate for fluctuations in the power input. Due to this rotation-readout a volumetric pump-characterisation is not necessary (as performed exemplary in Fig 2A to show linearity), as the rotation can be translated directly into piston movement. A maximal operation speed of around 150 rpm is achieved, which can be adjusted from -100% (counter-clockwise rotation) to 100% (clockwise rotation) and altered in discrete and integer values. The initial rotation speed is set via the ‘mymotor.Speed’ command in the MATLAB program (see S2 File). To guarantee a reliable dosage rate, the speed should be higher than ±5%. Furthermore, the maximal counter-pressure of the system should not exceed 1.4 bar, which was determined via a manometer. To stabilise the housing, cross struts can be installed if processes are performed with pressures reaching this limit.

In order to convey volumes in microliter scale, it was necessary to integrate gear-down modules in a daisy-chain-layout. The drastic reduction of rotation speed is mediated by up to two gearboxes connected in series. Each box consists of a worm-gear and a gearwheel with 24 teeth. Two boxes in a row lead to a reduction of the rotation speed and an increase in the running torque by the gear-down factor (*gdf*) of 24^2^. This enables an adequate speed of the linear actuator, which allows for movements of the syringe piston in micrometer scale over hours. An additional effect is the higher power transmission and resistance to the counter-pressure of viscous fluids. To generate a constant flux, a smooth and continuous movement of the syringe piston has to be ensured, which is guaranteed by the installed linear actuator. The length of the piston stroke of the linear actuator in the prototype is 40 mm, therefore, the volume that can be dosed is depending on the dimensions of the syringe inserted. An excerpt of setup-specific limits of flow rates adjustable at 100% motor speed ${\dot{V}}_{max}$, 5% motor speed ${\dot{V}}_{min}$ and the maximal total volume per syringe *V*_*total*,*max*_ can be found in Table 1 (data is shown in S2 File). To avoid effects with a fully retracted or extended piston, a distance of 35 mm should not be exceeded during dosing. Without gearboxes, this correlation *r*_*s*_ between the motor revolution and the distance covered by the linear actuator was 90° per millimeter during exponential feeding, and in consequence a piston movement of 1.11*10^−2^ mm per one degree of rotation. The *r*_*s*_ should be verified from time to time, because ageing or high counter pressures can affect this value (by applying extremal counter pressures over 1.5 bar the factor r_S_ changed irreversible to 150° mm^-1^). The addition of two gearboxes slowed down the piston movement to 1.11*10^−2^ mm per 576°. To minimise the standard deviation of the dosing process the distance of the piston movement should cover the whole range of 35 mm. This can be achieved by adjusting the syringe size as well as the concentration of the nutrient solution to the process requirements. The distance covered per volume was calculated based on the dimensions of the syringes. This value has to be determined for each syringe type individually, for instance for 10 mL syringes this parameter *l*_*V*_ is 8 mm mL^-1^ (see Table 1). It turned out to be beneficial to use a double sealing ring at the top of the syringe piston to reduce the friction during piston movement. For each volume *V*_*F*_(*t*) the corresponding degree of rotation *set*_*rotation*(*t*) which has to be executed at time point t, can be calculated as described in Eq 1.

$${set\_rotation(t)=V}_{F}(t)∙gdf∙r_{s}∙l_{V}$$ 1

**Table 1:** Specifications of the dosing unit. Depending on syringe size, gearboxes (0–2) and motor speed, different maximal flow rates (${\dot{V}}_{max}$), minimal flow rates (${\dot{V}}_{min}$), and total volumes (V_total, max_) can be accomplished. Flow rates are calculated based on a r_S_ of 150° mm^-1^ and a syringe-factor l_V_ = 1.8 mm mL^-1^ (for 50 mL syringes), l_V_ = 5 mm mL^-1^ (for 20 mL syringes), l_V_ = 8 mm mL^-1^ (for 10 mL syringes), or l_V_ = 57 mm mL^-1^ (for 1 mL syringes).

	${\dot{V}}_{max}$; mL min^-1^ 100% Motorspeed;				${\dot{V}}_{min}$; mL min^-1^ 5% Motorspeed			
Number of gearboxes	Syringe: 50 mL	Syringe: 20 mL	Syringe: 10 mL	Syringe: 1 mL	Syringe: 50 mL	Syringe: 20 mL	Syringe: 10 mL	Syringe: 1 mL
0	210.3556	75.7280	47.3300	6.6428	12.4212	4.4716	2.7948	0.3922
1	8.7648	3.155	1.9721	0.2768	0.5175	0.1863	0.1164	0.0163
2	0.3652	0.1315	0.0822	0.0115	0.0216	0.0078	0.0048	0.0007
*V*_*total*,*max*_; mL per syringe **35** mm (**l**_**V**_ mm mL^-1^)^-1^ = x mL	19.4444	7.0000	4.3750	0.6034	19.4444	7.0000	4.3750	0.6034

### Validation

As a first step in the validation of the LEGO-based dosing device, we tested different dosing profiles. Each experiment was performed in triplicates. First, the transformation of the motor speed to the volume dosed was examined. Therefore, the number of rotations was obtained via the ‘readRotation(mymotor)’ command of the MATLAB script for different motor speeds ranging from 0 to 100%. The rotations performed over a period of 10 seconds were translated into rotation speeds (° s^-1^) and setup-specific flow rates as described in S2 File and Table 1. Exemplary the resulting flow rates for 20 mL syringes are depicted in S2 File. As shown in Fig 2A a linear correlation between the rotation per second and the motor speed with the correlation factor of 8.5234° s^-1^ (%-motor speed)^-1^ was determined. Speed settings below 5% should be avoided because of an insufficient and non-linear response of the motor. Next, to show that even small volumes are reliably conveyed, a linear dosing profile at a rate of 10 μL h^-1^ was executed (Fig 2B). The pump performance is comparable to other micro-dosing systems available on the market. With a dosing rate of 10.16±0.07 μL h^-1^ in average the system presented in this work is in the same range of accuracy as the commercially available diaphragm pump Type 7615 (Bürkert).

Additionally, we evaluated an exponential dosing profile (Fig 2C) with different syringe dimensions (1 mL to 50 mL). An increase in the feeding rate over time, which here was exemplary chosen to be *a* = 1 h^-1^, could be precisely performed and was unbiased by the syringe dimension (1 mL—50 mL syringes). Therefore, the whole volume of the syringes (1 mL, 10 mL and 50 mL) was exponentially dosed over 5 h (Fig 2C). To determine the respective flow rates, the factor *b* = *V*_*F*_(5*h*)∙(*e*^*a*∙5*h*^)^−1^ was used to define an initial set point of volume. Using the feed-function *V*_*F*_(*t*) = *b*∙*e*^*a*∙*t*^, *b* is translating the total volume dosed over the whole experimental period of 5 h (in this case) into piston-movement. In all cases target flow rates were reached with an average oversupply of 2% to 4% (standard deviation of fit functions) for all syringe types. Finally, we demonstrated that the desired volumes dosed of the exponential feeds are correlating well with the actual volumes conveyed (Fig 2D). Taken together, this showed that the dosing system is reliably working, and can be used for linear as well as exponential feeds.

### Preparation of micro-encapsulated cells in alginate-beads

One example for a possible application of dosing techniques is the production of microcapsules, which is applied in many diverse fields of industry and biotechnology. The Grand View Research consulting company estimated in their report the size of the global microencapsulation market at USD 6.96 billion in 2017 [27]. The therefore produced capsules can be provided via diverse methods as coating, emulsion, spray technologies and dripping. Though, Tarun *et al*. identified as disadvantages of the so far patented microencapsulation techniques the costs and the need of sterilization among others [28]. The here presented dosing unit can reduce this two negative aspects at least for dripping technologies. The dosing procedure itself is a constant addition of solutions and therefore trivial. We tested the dosing unit for the production of microcapsules, which are applied in many diverse fields of industry and biotechnology. The production of uniform droplets for coating a solid or liquid phase is an example for a process in which a constant flow rate is crucial. This so-called microencapsulation finds its application in enclosing living cells in alginate-beads [29,30] and is a popular method to perform compartmentalisation of bioprocesses [31–34]. Not only the encapsulation of nutrients, e.g. in food or cosmetic industry, can be used for agent delivery, but also encapsulating microorganisms or enzymes is a smart tool for complex processes or bio-transformations, in which a two-phase-system or immobilisation is advantageous. In the production of microcapsules, two key factors have to be considered—the size of the microcapsules themselves and their size distribution. For some applications small particles are needed, whereas others require bigger sizes. Furthermore, a uniform size distribution often has a positive influence on the stability and reproducibility of the process, for instance rheological behaviour can be influenced by size [35]. Hence, during microcapsule production the aim is a narrow size distribution, which can be achieved by a suitable pump system, and the possibility to adjust the microcapsule size to the specific needs. The encapsulation of cells during biotechnological processes brings along a couple of advantages. It does not only seem to have cyto-protective effects, because the encapsulation in alginate beads protected the cells from stress and lengthened their shelf life [36], but it also enables an easy cell separation afterwards [37] or mediates cell retention in continuous processes [38].

**Fig 3:**
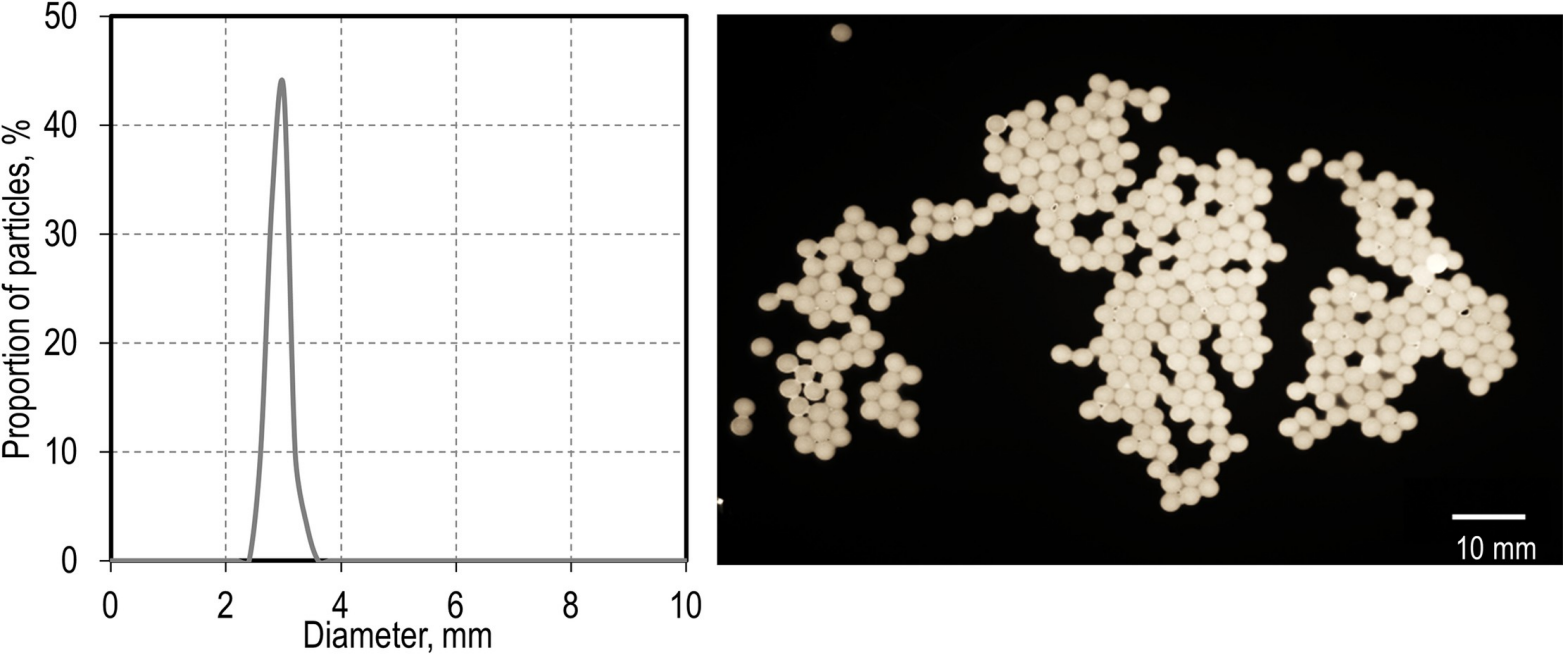
Encapsulation of *P*. *putida* into alginate beads. Left pannel: Size distribution of the microcapsules obtained using the dosing unit. Right: Light microscopy image of the particles produced.

Therefore, we aimed to encapsulate *Pseudomonas putida* EM178 in alginate beads to show the feasibility of this approach. An alginate-cell-mixture was dosed into a CaCl_2_ solution via the MINDSTORMS-based syringe pump with a flow rate of 0.2 mL min^-1^. As shown in the histogram depicted in Fig 3, the particles had a size distribution of 2.92±0.17 mm. The uniform shaped microcapsules have a smooth surface as seen by light microscopy and are enclosing a volume of around 13 μL as calculated from their diameter. This is an adequate volume to encapsulate living cells [39].

### Conceptual design of individual dosing profiles

Apart from a linear dosing as presented above, it is also possible to create complex dosing profiles with different flow rates. Therefore, the dosing function has to be divided into sub-functions. The principal procedure of how to provide a dosing function is described in the S3 File. As an example, in this work a linear ramp was used to boot and to cool down the flow rate. This type of flow-profile can be used to change buffers gradually for example to protect chromatography columns against the precipitation of salt. Here, a profile (shown in S3 File) with two linear changes in the flow rate *f*(*t*) and h(*t*) was performed. The function *g*(*t*) describes a constant flow rate over time. The number of rotations needed for the dosing was calculated taking into account the phase of the profile. To determine the volume already dosed (which can be transformed via Eq 1 into conducted rotations) for different time points during the first phase, *f*(*t*) are integrated from *t*_0_ to the actual time *t*. In the second phase, the already dosed volume of phase one (*t*_0_−*t*_1_) is summed up with the volume of second phase *g*(*t*) from *t*_1_ to the actual time *t*, and so on. The general procedure is shown in S3 File. These considerations allow to create complex and individual dosing profiles for a variety of applications. The designed dosing profiles can be integrated into the MATLAB script by adapting the specific feed function ‘set_rotation’. For more information how to modify the function in the script ‘set_rotation’ please refer to S1 and S3 Files. The workflow of an exemplary application for complex and combined dosing profiles is introduced in the next chapter.

### Establishing a dosing profile for protein purification

Another field of application for automated pump systems can be found in the downstream processing. Many capturing and polishing stages are including washing steps or elution profiles, which can be achieved by employing a controllable pump system. For instance, the separation of his-tagged proteins from cell lysate usually takes place via immobilised metal-affinity chromatography (IMAC) using an imidazole gradient driven elution [40]. For the purification process it is desirable to combine the application of a gradient with a homogeneous flux, which avoids damage of the column-matrix and enables a uniform elution. By this a reliable performance in the separation process is achieved [41].

**Fig 4:**
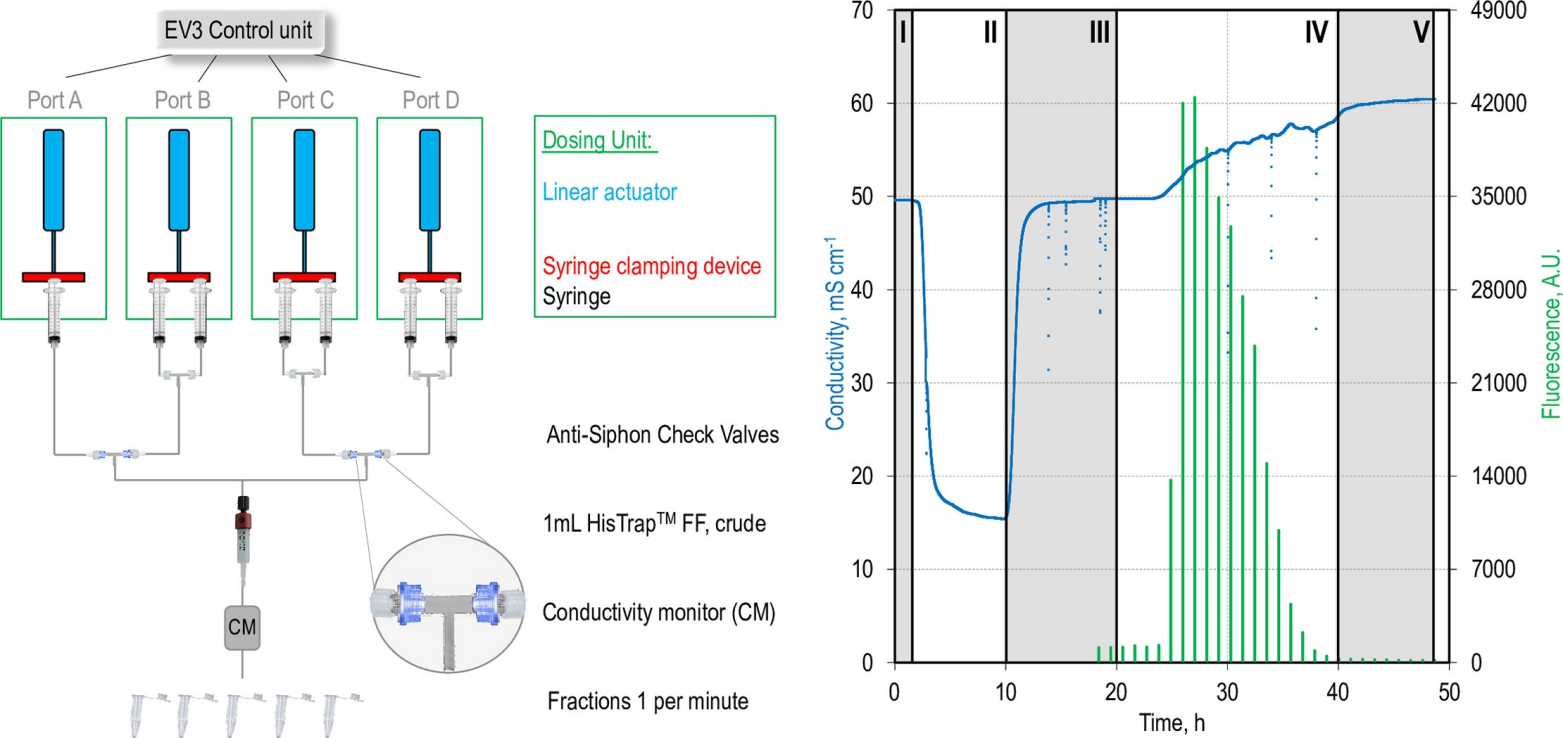
By connecting four dosing units to the programmable LEGO-MINDSTORMS EV3 brick a protein purification process was performed. The schematic setup shown on the left was used to dose the sample (port A), the wash- (port B), and elution-buffer in two concentrations (port C and port D) to perform an IMAC process according to the column-manufacturer's specifications. As depicted on the right, after a dead-volume caused delay (I), the loading of the sample (II), the washing procedure (III), the gradient elution (IV), and the rinse with 100% elution buffer (V) can be observed in conductivity (blue). Protein occurrence was demonstrated via measurement of the green fluorescence signal in fractionated flow through.

**Fig 5:**
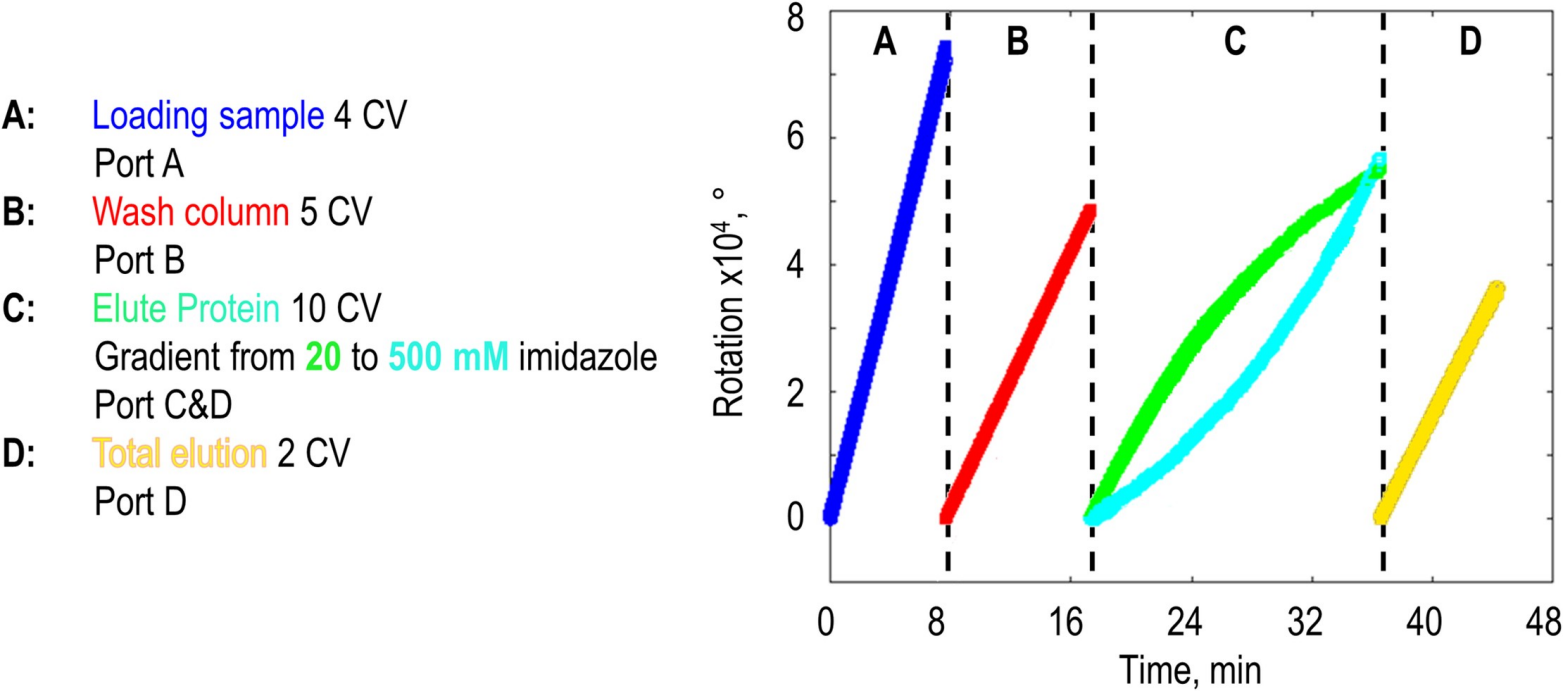
The degrees of rotation performed by the motors of the dosing units connected via port A to D with the programmable. MINDSTORMS-**EV3 Brick.** The purification protocol consists out of four steps. During step A the dosing unit in port A (blue) was loading four column volumes (CV) of cell lysate to the His-Trap column. In step B a wash buffer (20 mM Imidazole) was conveyed from two syringes via the dosing unit in port B (red). Starting with 20 mM Imidazole and ending with 500 mM Imidazole a gradient was dosed in step C, controlled simultaneously by an increasing flow rate via port D (cyan) and a decreasing flow rate performed by port C (green). To guarantee total protein elution in step D two CV of 500 mM Imidazole buffer were dosed via port D (yellow).

Therefore, we tested the dosing unit for the application in protein purification. We established an automated protein purification process for his-tagged proteins (eGFP) by combining different dosing profiles as it can be performed via the ÄKTA chromatography system from GE Healthcare Life Sciences (GE). Besides the ÄKTA-system, automated purification processes can be conducted in other commercial available chromatographic systems as well. Though they can cost 10000–50000 € even in the standard configuration. Alternative methods to the automated laboratory equipment are spin traps or manually loadable columns. Because these protocols contain besides a binding and a washing step, an elution step as well, these systems are elaborately and much more limited in capacity and performance as automated systems. As schematically depicted in Fig 4 (left), four dosing units were connected to a His-Trap column and a conductivity monitor. The cell lysate containing the eGFP protein was loaded onto the column and throughout the whole process every minute a fraction was collected and its conductivity as well as green fluorescence was determined. As can be seen in Fig 4, there is a delay in the occurrence of the signal, which can be attributed to the dead-volume of the system (Fig 4; right, I). During the loading process a decrease in conductivity is observed (Fig 4; right, II), which is caused by the charge of the cell lysate. In the subsequent washing step the unbound protein was removed from column, which is indicated by the approximation of the conductivity signal to the regular level of the wash buffer (Fig 4; right, III). In the elution step a linear imidazole gradient is applied by simultaneous feeding from two ports (C and D) equipped with different imidazole concentrations with changing convey rates. By feeding a constant volume in total, the linear gradient was achieved via a decrease of the flowrate of low concentrated imidazole dosed by port C and simultaneous increase of the dosage of the high-concentrated imidazole solution, which was provided by port D. During the elution process the increase in imidazole concentration is reflected by the rise of conductivity (Fig 4; right, IV). The successful elution of the purified protein eGFP was demonstrated by measuring the green fluorescence in the fractionated flow through. For processes with additional fittings, pressure can exceed the maximum the system can handle. At the end of the process, a constant flow of 100% elution buffer was performed for 2 column volumes (CV), to ensure a complete elution (Fig 4; right, V). Effectively, no further GFP-Signal was detected after elution. Therefore, by employing the LEGO-based dosing unit the time-consuming purification procedure could be performed automatically, according to conventional protocols as provided from GE. The specification of the process can be found in S1 File. The sum of rotations diagram of all steps was depicted in Fig 5.

### Dosing profile for biotechnological feeding strategies

In biotechnological cultures, besides the solved oxygen concentration, mostly the availability of the carbon source is the growth limiting factor. During semi-continuous operation-modes, as it is the case for an exponential feeding strategy, the bioreactor system with the volume *V*_*R*_ has to be supplemented by a continuous or at least intermittent substrate dosing with a specific influx ${\dot{V}}_{in}$ and a substrate concentration of *c*_*S*0_ in the feed. The actual substrate concentration *c*_*S*_ in the cultivation vessel can be described via the mass balance which includes the incoming substrate ${{\dot{V}}_{in}∙c}_{S0}$ and the metabolisation of substrate *c*_*X*_∙*q*_*S*_∙*V*_*R*_ [42] shown in Eq 2 (in this case *q*_*S*_ is the substrate uptake rate, mg_Substrat_ mg_CDW_^-1^ h^-1^).

$$\frac{d(V_{R}∙c_{S})}{dt}={\dot{V}}_{in}∙c_{S0}-{c_{X}∙q}_{S}∙V_{R}$$ 2

Under substrate-limiting conditions and assuming that the substrate is consumed immediately, it can be expected that *c*_*s*_≪*c*_*s*0_. Furthermore, the energy demand of *E*. *coli* for maintenance was very low (non-growth-associated-maintenance: 8.39 mmol_ATP_ g_CDW_^-1^ h^-1^ [43]) and can therefore be neglected [11]. By limiting the availability of carbon-source based on the biomass yield *Y*_*XS*_ and the initial biomass *c*_*X*0_ the flow rate ${\dot{V}}_{in}(t)$ of substrate needed for growth rate set (μ_*set*_) can be calculated time dependent as described in Eq 3. We simplified the equation assuming of a constant volume, even though during processes with an exponential feeding strategy the volume in the bioreactor is increasing, because the volume dosed over the whole process was in the same range as the overall volume of the samples collected.

$${\dot{V}}_{in}(t)=\frac{\mu_{set}∙V_{R}∙c_{x0}}{Y_{XS}∙c_{s0}}∙e^{\mu_{set}∙t}$$ 3

To determine the volume dosed for each point *t* the flow rate ${\dot{V}}_{in}(t)$ was integrated over time leading to Eqs 4.A and 4.B. This growth specific dosing profile over time can be formulated in two ways, depending on whether the total volume dosed (Eq 4.A) or the total rotations of the motor (Eq 4.B), derivation can be found in S5 File) is considered.

$$V_{F}(t)=\int_{0}^{t}{\dot{V}}_{in}(t)=\frac{V_{R}∙c_{X0}}{{Y_{XS}∙c}_{S0}}∙(e^{\mu ∙t}-1)$$ 4.A

$$set\_rotation(t)=\frac{V_{R}∙c_{X0}}{Y_{XS}∙c_{s0}}∙(e^{\mu_{set}∙t}-1)∙gdf∙r_{s}∙s_{V}$$ 4.B

**Fig 6:**
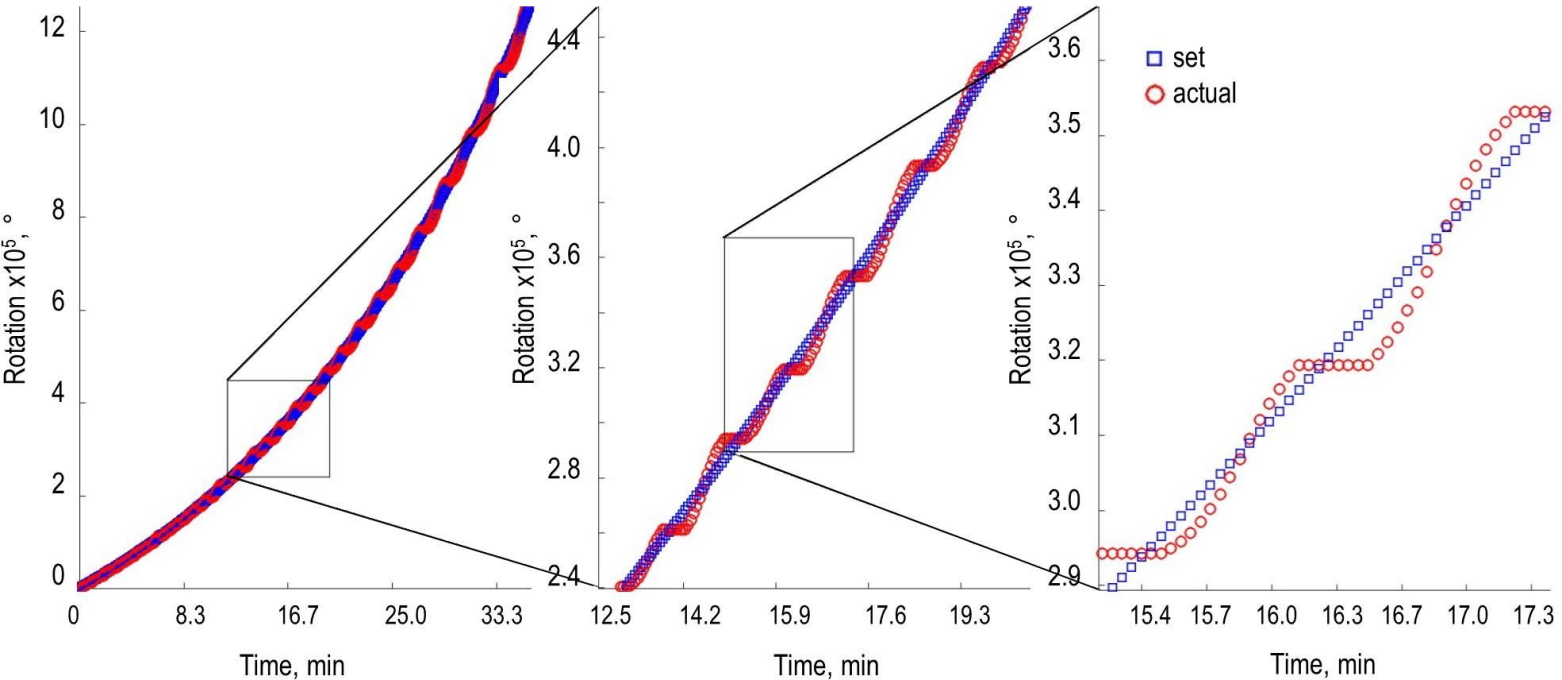
The actual sum of rotation is oscillating around the set number of rotations. To accomplish adequate small steps and guarantee a continual flow the actual sum of rotations is oscillating around the set number of rotation. As shown in the zooms, the fluctuations of the exponential feeding profile are in the scale of a few seconds.

As shown in Fig 6, the actual rotation typically was oscillating around the set rotation, which resulted in a feed that on average was correctly. Deviations of the rotation from the set point were corrected within seconds by the MATLAB script. We assumed that this inaccuracy of dosing is negligible as it is not in the same time scale as the adaption of the cellular metabolism.

### Setting the growth rate in parallel shaking flasks

**Fig 7:**
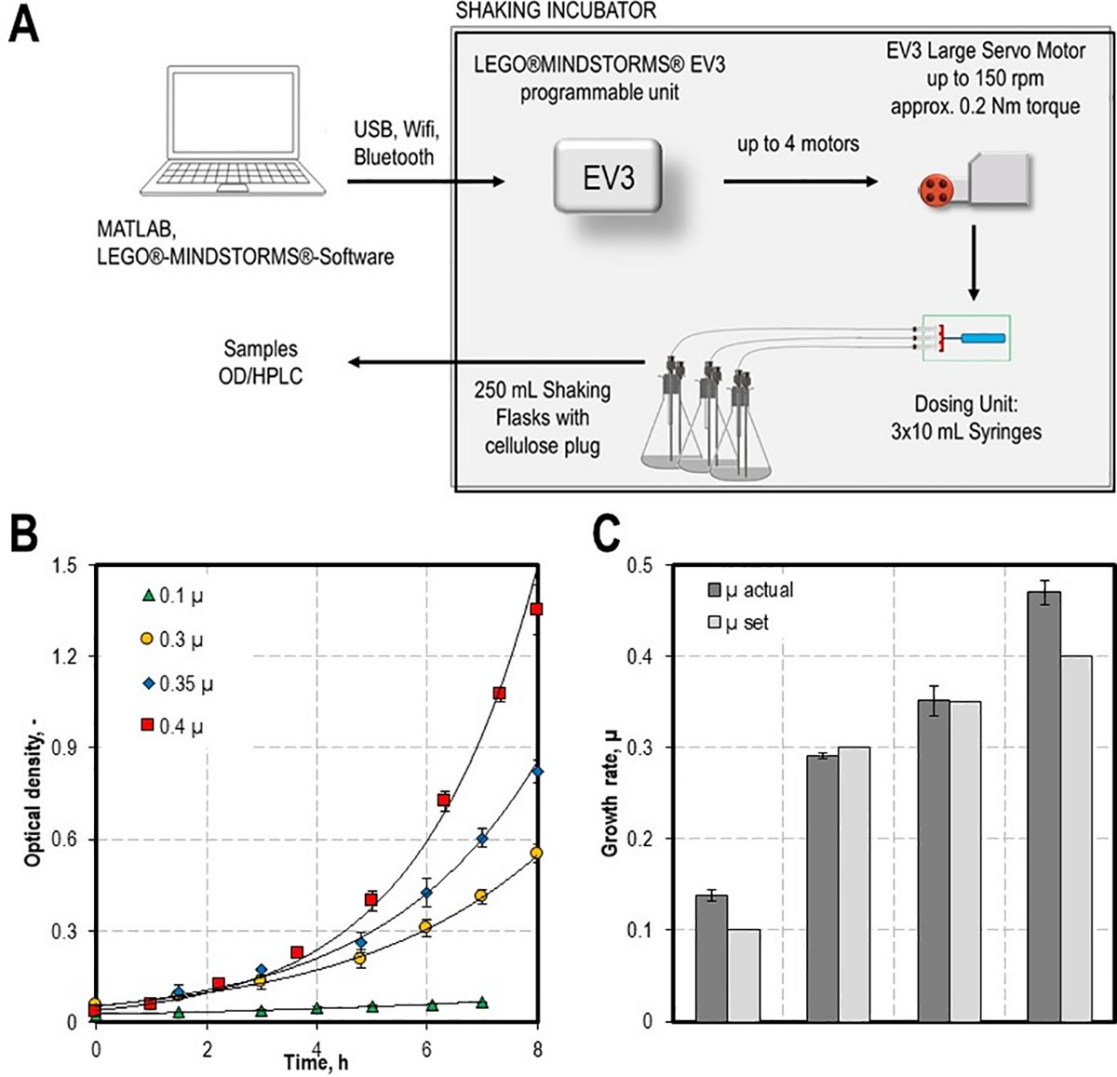
Setting exponential growth rates of bacterial cultures in the range of 0.1 to 0.4 h^-1^ by applying a C-source limited feeding strategy. The experimental set-up is shown in (A). The strain specific input variables for the dosing unit, like biomass-substrate yield or the desired growth rate were adjusted in the MATLAB script (S1 File). Calculated rotations are transduced from the EV3 Brick to the large EV3 motor. The LEGO-syringe pump is translating this rotation into a piston movement. The shaking flasks were sealed with sterile cellulose plugs and the feeding solution was dosed directly into the medium. A Bluetooth-connection enabled the wireless transmission of signal into the shaking incubator. Samples of the *E*. *coli* cultures were taken manually via a second cannula. Optical density as well as glucose concentration was determined. (B) Optical density of the cultures with the different growth rates set. (C) Comparison of the actual growth rate measured (μ actual) with the theoretical growth rate set (μ set).

In order to test, whether the constructed dosing unit is beneficial for parallel cultivations in shaking flasks with a defined growth rate, we conducted the experiment sketched in Fig 7A. The dosing unit was equipped with syringes containing the C-source glucose and was programmed to implement an exponential feed into the shaking flasks. Because no C-source was in the medium the feed was started directly after inoculation. Therefore, the MATLAB script (S1 and S6 Files) was used to translate the process parameters into rotations and consequently to dosed substrate. To guarantee an easy handling the dosing unit was fixed inside the shaking incubator and the feed was dispensed into the shaking flasks via tubes. Cannulas transported the feed directly into the medium to ensure a continuous flow. We set different growth rates in the range of 0.1 to 0.4 per hour by adjusting the nutrient flow (see Table 2) and measured the optical densities and the glucose concentration of the cultures. Samples were manually collected via a second cannula. An exponential increase in the optical density was observed for all cultures (Fig 7B) and the actual growth rates matched the desired ones very well (Fig 7C). By comparing the actual growth rates (μ_actual_) with the envisaged growth rates (μ_set_) (Fig 7C), an average discrepancy of 13.2% between μ_actual_ and μ_set_ was observed. To verify that the growth of the cells is limited by the substrate availability, the glucose concentration in the medium was determined via HPLC. Comparing a regular batch-process at maximal growth rate with exponentially feeded cultures (S1A Fig), the glucose was only detectable in traces (mg L^-1^ scale) (S1C Fig), which shows that the substrate was completely consumed. The typical accumulation of side products which usually occurs in batch processes (S1B Fig) was reduced to mg-scale (S1C Fig). Only, the overflow products acetate and lactate were detectable in some of the samples, however no continuous increase was observed. We assume that this is due to re-metabolisation of the side products by the cells as a consequence of the carbon limitation. This is of advantage, when mass balances should be closed or product yields enhanced.

**Table 2:** Adjusted growth rates and parameters used for performing growth experiments with *E*. *coli* HMS174/DE3. Depending on growth rate and initial biomass concentration volume was kept in dosable range by modifying the substrate concentration of the feeding solution and the syringe type.

Growth rate set, μ h^-1^	Initial biomass, c_X0_ mg mL^-1^	Substrate concentration feed, c_S_ mg mL^-1^	Syringe type
0.1	0.03	36	1 mL
0.3	0.05	18	10 mL
0.35	0.05	18	10 mL
0.4	0.04	36	10 mL

### Controlling the substrate uptake rates to determine the cost of a heterologous load

**Fig 8:**
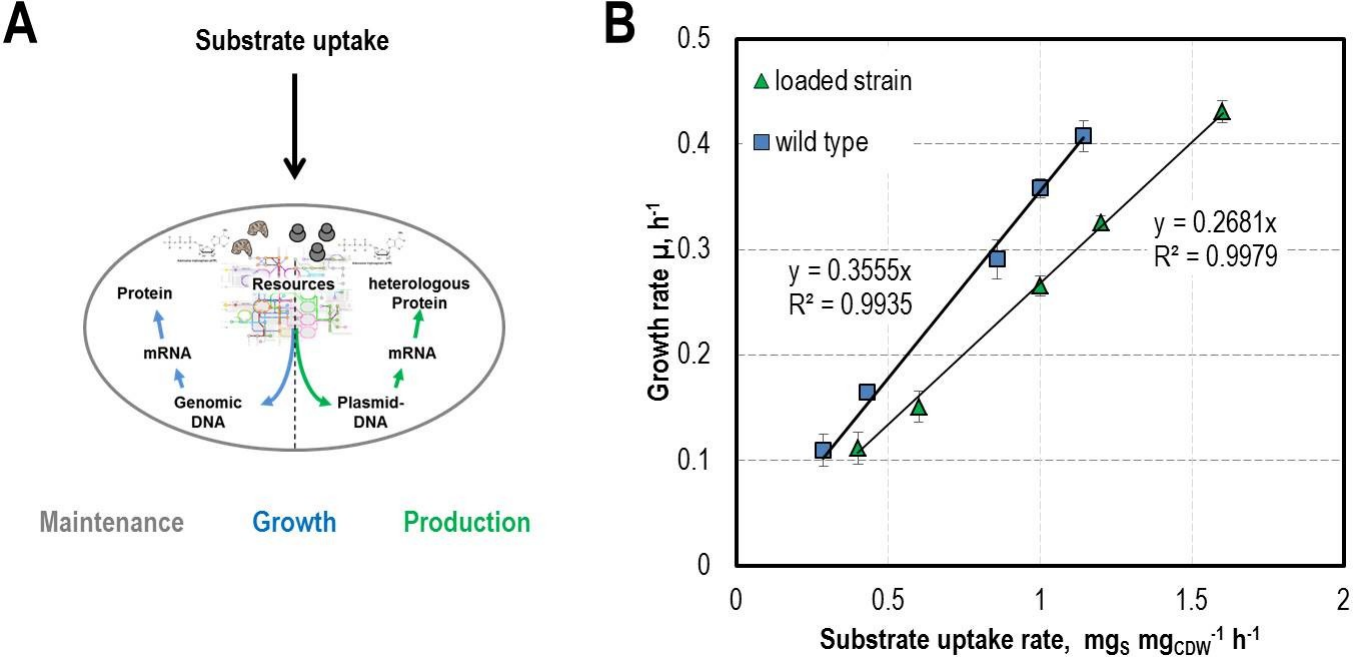
Energy demand in strains carrying a plasmid with a heterologous load. (A) In engineered host cells the energy provided by the substrate has to be divided between regular tasks and a heterologous load. (B) Comparison of the growth rates reached by the wildtype and a plasmid bearing strain at defined substrate uptake rates. The difference in the growth rates reached with the same substrate uptake rate can directly be attributed to the altered genetic setup. Shown are the means of at least three independent cultures.

The dosing unit is not only able to replace costly commercial systems but also to serve a possible solution for an actual hot-topic question in biotechnology, namely the energy demand of a cell [14,44,45]. We set out to determine the energy demand for a heterologous load by using the LEGO-MINDSTORMS-based dosing unit. Because the C-source is the growth-limiting factor, substrate uptake rates q_S_ < q_Smax_ can be adjusted by the feed. If the supply of the C-source does not keep pace with the maximal substrate uptake rate, cells perform a modified uptake q_S_ adapted to the amount of provided C-source. Under the assumption that all dosed molecules are consumed immediately, the rate of providing nutrition should be a measure for substrate uptake rate and therewith for growth rate. In wildtype cells the substrate taken up is mainly used for growth and maintenance of the viability. For *E*. *coli* cells the energy demand for the non-growth-associated-maintenance (NGAM) is in the magnitude of 8.39 mmol_ATP_ g_CDW_^-1^ h^-1^ [13] and therewith in a negligible range. In the case of cells carrying a plasmid with a heterologous load, the energy provided by the substrate has to be divided between regular processes and the heterologous production (Fig 8A). The specific biomass yield Y_XS_ is defined as the quotient of the growth rate and the substrate uptake rate. It is a proxy for the part of the energy that is invested into growth. When the cells are carrying a plasmid, they reach lower levels of biomass as compared to unloaded cells with the same uptake rate. This relation was analysed by growing *E*. *coli* HMS174/DE3 wildtype and the same strain carrying the plasmid pTRA-51hd [46]. Via dosing defined substrate uptake rates the related growth rates were determined. As shown in Fig 8B, the growth rates of the loaded strain were lower compared to the wildtype strain at the same substrate uptake rate. This is caused as a part of the energy was needed for plasmid related processes. The corresponding biomass yields Y_XS_ can be extracted from the slopes of Fig 8B much more reliable than—as it is common—out of one single μ-q_S_-relation. The total energy demand of the loaded cells can be described as the sum of the energy demand for growth q_S,μ_ and for the additional load (q_S,load_) (Eq 5.A). The energy demand for the load (q_S,load_) was calculated to be 33% via Eq 5.B (the derivation can be found in S5 File). This results in a total energy uptake-rate for the plasmid loaded strain (q_S,total_) of 133% of the unloaded wildtype strain in order to accomplish the same growth rate. Because of the straight linear correlation, the percentage of the extra energy demand for the loaded strain seems to be unaffected by growth rate.

$$q_{S,total}=q_{S,\mu }+q_{S,load}$$ 5.A

$$q_{S,load}=q_{S,\mu }∙\frac{Y_{XS,WT}-Y_{XS,loaded}}{Y_{XS,loaded}}$$ 5.B

Therefore, we could show that by controlling the substrate uptake in a process via limiting the substrate availability with an exponential feed not only the additional energy demand for different constructs can be calculated but that it is also possible to estimate the biomass yield very precise.

## Materials and methods

### Assembling the dosing unit

The dosing unit consists of 269 LEGO-bricks which are listed in S1 Table. It can be rebuilt easily with the included manual. After building up the basic structure, the gearboxes were added or removed in order to vary the translation to the motor and thus the scale of the dosed volume, which ranges from microliter up to millilitre per minute. For the dosing of volumes in a microliter scale, e.g. the feeding of a carbon source into a cell culture, two gearboxes were used, whereas for flow rates in mL-per-min-scale no or just one gearbox was needed. The servo motors used were connected to the plug-ins A to D of the EV3-inelligent brick. As described in detail in S1 File the MATLAB software was connected to the EV3-brick as well as to the motors. Briefly, after installing MATLAB Support Package for LEGO-MINDSTORMS-EV3 Hardware (version 18.1.0.0, by MathWorks MATLAB Hardware), the control unit was connected to MATLAB with the legoev3 command. The MATLAB script consists of two parts. The ‘*set_rotation*’ feed function, which determines the theoretical rotations that are performed until the specified time ‘tt’, and the script which adjusts the motor speed. For detailed operating instructions see S1 File.

### Integrating the dosing unit into experimental setups

To perform processes, which require sterile conditions, the following procedure was carried out under a laminar flow bench. Depending on the volume dosed, a suitable set of syringes was selected. In this work, all processes were performed either with 1, 10, or 50 mL syringes. The liquid of choice was soaked into the single-use syringes, which had been previously connected to an autoclaved tube system. The loaded syringes were inserted into the housing of the dosing unit, as described later (syringe insertion). After the fixation into the syringe clamping device, the tube system was connected via Luer-lock to the target vessel either with a cannula or directly to the micro-columns. Finally, the feed function of interest was adjusted and the dosing script was started.

### Determination of dosed volume

The transferred volume was gravimetrically determined. Therefore, droplets were collected in a vial (Macherey-Nagel, Vial N9-1.5, GW, k, 11,6x32, flat, SF, Ref-Nr: 702283), which was sealed by a cap with a PTFE septum to minimize evaporation. The volume, which was dosed into the vial via a cannula-connected hose, was weighed (Sartorius analysis scale Extend ED124S) by subtracting the weight of the vial. The volume was calculated based on the density of water (997 kg m^-3^) and the relation 1 L = 1 dm^3^.

### Dosing profiles

The feed function was varied depending on the corresponding dosing profile. For a constant flow rate, a motor speed between 5% and 95% was set manually. For more complex dosing profiles, in defined intervals (in case of the protein purification protocol every 0.001 seconds) the amount of rotations executed was compared to the theoretical number of rotations needed to provide the desired flow. This was calculated by the ‘*set_rotation*’ feed function. If the number of measured and calculated rotations differed, the motor speed was adjusted to correct the difference. A general procedure to calculate feed functions is shown in S3 File on the example of a constant feeding profile with an initial mounting process of the flux and a short cool down.

### Exponential feeding profile

In order to achieve an exponential feeding profile, the actual rotation of the motor had to be aligned to the theoretical rotation needed to supply the desired flow. This theoretical rotation is named ‘*set_rotation*’ in the MATLAB script (S1 File) and was calculated every 30 seconds. The starting motor performance was set to 5% to ensure a reliable dosing. The motor speed was adjusted to ±1% in case the actual number of rotations did not match the theoretical rotation needed. Growth rates between 0.1 and 0.4 h^-1^ were accomplished by choosing substrate concentrations of 18–36 mg mL^-1^ (Table 2), an initial concentration of biomass of around 0.04 mg mL^-1^, and a starting volume of 50 mL as described in S1 File. The biomass yield *Y*_*XS*_ = 0.35 mg_CDW_ mg_Substrate_^-1^ for wild type strain *E*. *coli* HMS174/DE3 (S4 File) was used as a parameter in the MATLAB script. The experiments were performed in triplicate per dosing unit in parallel. Therefore, three 10 mL syringes with a length-per-volume factor l_V_ of 8 mm mL^-1^ were used. The r_S_ factor (rotations in degree per length) for the dosing unit was set to 90° mm^-1^. To convey volumes on microliter-scale two gearboxes were integrated to slow down the rotation.

### Cultivation setup and metabolite quantification

The *E*. *coli* species used in this work was the strain HMS174/DE3 [47]. To provoke a burden (shown in Fig 8), the inducible plasmid pTRA-51hd was inserted [46]. All pre-cultures of *E*. *coli* cells were cultivated first in lysogeny broth (LB) medium [48] and then in minimal medium [46] amended with trace elements, and 20 mM of glucose as C- source. The pH was set to 7.4 (25°C) and a salt solution as well as trace elements were added. Pre-cultures were grown the same way as described in [46], before they were used to inoculate 50 mL of the minimal medium mentioned above without C-source to a cell-dry-weight-concentration of around 0.04 mg mL^-1^. The principle cultivation setup is depicted in Fig 7A. The cultivation took place in sterile unbaffled 250-mL-shaking flasks at an agitation frequency of 250 rpm (5 cm eccentricity) and a temperature of 37°C. The C-source was added with the MINDSTORMS-based feeding system. The syringes were connected to sterile platinum-treated silicone hoses (30 cm length, inner diameter 0.5 mm, CH23.1, CarlRoth) with Rotilabo-Luer-connectors (CT58.1 and CT62.1, Carl Roth) and subsequently filled with the substrate solution with a concentration (c_S0_) of 18 to 36 mg mL^-1^. To avoid un-continuous dosing by droplet formation, cannulas (neoLab, Luer-Lock connection, 1.0 x 200 mm, SKU: 2–3113) were used to convey substrate via the Platinum-treated silicone hose directly into the medium and shaking flasks were sealed with a cellulose plug. These plugs were pierced with two cannulas (one for sampling, one for dosing). In periodic intervals throughout the growth phase 1 mL samples were taken for analysis. The optical density (measurement wavelength: 600 nm, measurement bandwidth: 9 nm, number of reads: 25, Settle Time: 0 ms) was determined every 1.5 h in a plate reader (Infinite 200, Tecan) and the metabolites were quantified via the Agilent 1100 HPLC systems (Agilent). For the analysis, 0.8 mL were taken as a sample and centrifuged at 4°C and 13000 rpm (Microcentrifuge 5418, Eppendorf). After adding 0.01% (w/v) EDTA, the supernatant was filtered via a 96-Mulit-well plates (0.2 μm, AcroPrep Advance 96 Filter Plate, Pall Corporation). The Shodex SUGAR SH1011 (Phenomenex) was used as a chromatography column to separate metabolites. 20 μL of samples were analysed with 0.5 mM H_2_SO_4_ as the mobile phase with a flow rate of 0.45 mL min^-1^ and 30°C column temperature. The quantification was performed via RI-detected peak areas with regard to a concentration-standard. Spectras were manually integrated with the ChemStation software (Agilent).

### Cell encapsulation in alginate beads

To encapsulate *Pseudomonas putida* KT2440 EM178 [49] cells into alginate beads, up to 3% (w/v) of Na-alginate was added to 10 mL of a bacterial culture with a cell-concentration of 4 mg mL^-1^. Via the septum, the solution was dosed with the help of a Sterican cannula (Luer-Lock 0.80x40 mm, size 2, green) into a sterile 250 mL bottle (Schott) filled with 50 mL of a 50 mM CaCl_2_ solution. Droplets gelled under moderate stirring in the CaCl_2_ solution. The alginate-solution was dosed with a 10 mL syringe using a constant flow rate of 0.2 mL min^-1^ (100% motor speed and a *gdf* of 576). No feed function was required for running this constant flow rate. All steps for the assembly of the system were performed in a sterile atmosphere with sterile solutions and consumables.

### Protein purification

The purification of a his-tagged protein in general takes place via an IMAC procedure. As an example, recombinantly expressed eGFP was purified out of a pre-filtered (syringe filter Minisart High Flow, pore size exclusion 0.22 μm, Sartorius) cell lysate after cells were disrupted via a high pressure homogeniser. To trap the protein, a 1 mL- column (GE Life Sciences, 11-0004-58 HisTrap FF Crude) was used. A four-step dosing profile executed by four dosing units in port A to D, which were controlled simultaneously via a MATLAB-script (S1 File), was conducted. The process was performed at a flow rate of 0.5 mL min^-1^. Sample, wash buffer (20 mM sodium phosphate, 0.5 M NaCl, 20 mM imidazole, pH 7.4), and elution buffer (20 mM sodium phosphate, 0.5 M NaCl, 500 mM imidazole, pH 7.4) were provided in 10 mL syringes (HSW SOFT-JECT, 10(12) mL, Luer, 5100.X00V0; l_V_ = 5 mm mL^-1^), which were connected with platinum-treated silicone hoses (up to 137 cm length per dosing unit, inner diameter 0.5 mm, CH23.1, CarlRoth) and Rotilabo-Luer-connectors (CT58.1 and CT62.1, Carl Roth) to the column. To avoid back-mixing, check valves (Nordson MEDICAL, Female Locking Luer to Male Locking Luer, SCV06252) were installed at the intersections. The purification procedure was performed as recommended by the column manufacturer. As shown in Fig 5, first 4 column volumes (CV) of pre-filtered lysate were applied on the column by port A (1 syringe). This was followed by a 5 CV wash step from port B (wash buffer; 2 syringes; therefore, flow rate was halved). To elute the protein bound, a gradient between the 20 mM imidazole wash buffer and the 500 mM elution buffer was established via port C and D (wash buffer C; elution buffer D; 2 syringes each; therefore, flow rate was halved) over 10 CV. To guarantee a total elution 2 CV of elution buffer were conveyed afterwards from port D (2 syringes; therefore, flow rate must be halved). By connecting a conductivity monitor (C9n, 29-0113-63, GE) in series with the column the performed gradient was monitored. The difference in imidazole increased the conductivity from 50 to 60 mS cm^-1^. During step C and D fractions were manually sampled every minute and the fluorescence of 200 μL was analysed in a microtiter plate reader (Infinite 200, Tecan; fluorescence: 485/520 nm; Excitation Bandwidth: 9 nm, Emission Bandwidth: 20 nm, ReadingMode: Top, Lag Time: 0 μs, Integration Time: 20 μs, Number of Reads: 25, Settle Time: 0 ms, Gain Value: 50).

## Supporting information

S1 Table Used LEGOBricks. The name and the part number of the used LEGOBricks is depicted here.
(XLSX)

S1 Fig Comparison of extracellular metabolites during batch and exponential feed. (A) maximal growth rate in batch process and during exponential feed. (B) Acetate production in batch process. (C) Accumulation of fermentative side products during exponential feed.
(PDF)

S1 File MATLAB scripts. Scripts for performing a protein purification and for a fed-batch process.
(PDF)

S2 File Transformation of motor speed into flow rates. S2 Table depicts the calculated values for the transformation of motor speed into flow rates. S2 Fig shows (A) a picture of one dosing unit connected to the EV3 brick and (B) the resulting flow rates for 20 mL syringe for different motor speeds.
(PDF)

S3 File General derivation of dosing functions. S3 Fig (A) Define functions of the dosing profile, (B) Calculated formulas, (C) Calculation of complex dosing profiles and (D) the actual sum of rotations.
(PDF)

S4 File Derivation of dosing function for fed-batch processes. Parameters and Equations for the derivation, the figure shows the C-source consumption over cell dry weight production.
(PDF)

S5 File Calculation of the energy demand of heterologous load. The distribution of cellular resources provided by substrate uptake is shown.
(PDF)

S6 File MATLAB files ‘Feed’ and ‘Protein purification’. (ZIP)

S7 File Abbreviations and variables. (PDF)

S8 File Raw data. (ZIP)
