# Supplementary material for: An automated and parallelised DIY-dosing unit for individual and complex feeding profiles: Construction, validation and applications
Source: PLoS One. 2019 Jun 19;14(6):e0217268. doi: 10.1371/journal.pone.0217268 (PMC6583958; doi:10.1371/journal.pone.0217268)
Supplement: S1 Fig — (A) maximal growth rate in batch process and during exponential feed. (B) Acetate production in batch process. (C) Accumulation of fermentative side products during exponential feed. (PDF) [file pone.0217268.s002.pdf]

Supporting Information 4

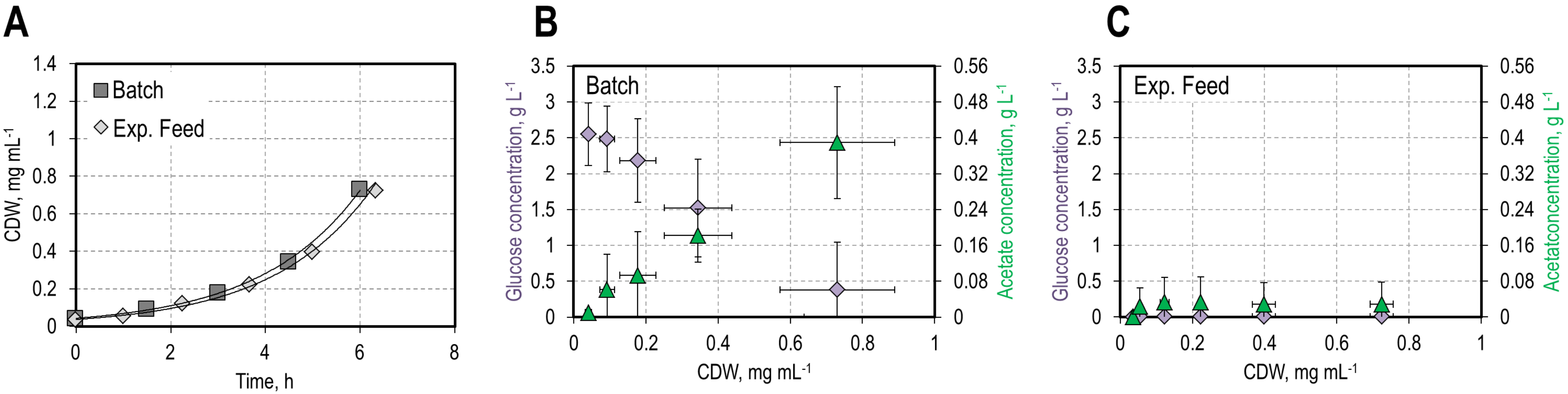

Fig S4: Comparison of extracellular metabolites during batch and exponential feed. As shown in (A) the maximal growth rate in batch process was adjusted in a second cultivation by an exponential feed. During glucose consumption acetate was produced as an overflow product in batch process (B). Keeping the substrate availability limited via an exponential feed, a negligible accumulation of fermentative side products was observed (C).
