# Supplementary material for: An automated and parallelised DIY-dosing unit for individual and complex feeding profiles: Construction, validation and applications
Source: PLoS One. 2019 Jun 19;14(6):e0217268. doi: 10.1371/journal.pone.0217268 (PMC6583958; doi:10.1371/journal.pone.0217268)
Supplement: S1 File — Scripts for performing a protein purification and for a fed-batch process. (PDF) [file pone.0217268.s003.pdf]

Supporting Information 1

To use the third-party-software MATLAB in combination with the EV3-MINDSTORMS -Block you can find commands at the mathworks support centre. Before adjusting the following test program, please read instructions at https://www.mathworks.com/help/supportpkg/legomindstormsev3io/index.html and install the MATLAB Support Package for LEGO-MINDSTORMS EV3 Hardware.

Performing a protein purification

The purification protocol consists out of the four steps A to D (load, wash, elution, and total elution):

1. Save functions ‘set_rotationA.m‘ for step A as a separate MATLAB file and adjust parameter of interest:

```
function y = set_rotationA(tt)

%%% PARAMETER MUST BE ADJUSTED %%%
load=8*60; % s to loading sample
wash=10*60; % s to wash sample
elute=20*60; % s to elute by gradient from 0% elution buffer to 100%
total_elute=8*60; % s to elute with 100% elution buffer
Vset=0.5/60;        % mL/sec flow rate (one syringe!)
lv = 5;           % mm/mL, length per volume for syringe
rs = 150;         % degree/mm rotation in degrees per length
gdf = 24;          % -, gear-down factor, one gearbox

t0=0;
t1=load
t2=load+wash
t3=load+wash+elute
t4=load+wash+elute+total_elute
Vt  = Vset*(tt-t0);% integrated V dosed between t0&t1
y = Vt*lv*rs*gdf % °, degrees of rotation

plot(tt, y,'bo')
xlabel('time, s');
ylabel('rotation, #');
legend('rotation set')
hold on
end
```

2. Save functions ‘set_rotationB.m‘ for step B as a separate MATLAB file and adjust parameter of interest:

```
function y = set_rotationB(tt)

%%% PARAMETER MUST BE ADJUSTED %%%
load=8*60; % s to loading sample
wash=10*60; % s to wash sample
elute=20*60; % s to elute by gradient from 0% elution buffer to 100%
total_elute=8*60; % s to elute with 100% elution buffer
Vset=0.25/60;        % mL/sec flow rate (two syringes!)
lv = 5;           % mm/mL, length per volume for syringe
rs = 150;         % degree/mm rotation in degrees per length
gdf = 24;          % -, gear-down factor, one gearbox

t0=0;
t1=load
t2=load+wash
t3=load+wash+elute
t4=load+wash+elute+total_elute
Vt=(Vset*(tt-t1);% integrated V between t1&t2
y = Vt*lv*rs*gdf % °, degrees of rotation

plot(tt, y,'ro')
xlabel('time, s');
```

```
ylabel('rotation, #');
legend('rotation set')
hold on
end
```

3. Save functions ‘set_rotationC.m‘ for step C as a separate MATLAB file and adjust parameter of interest:

```
function [y,z] = set_rotationC(tt)

%%% PARAMETER MUST BE ADJUSTED %%%
load=8*60; % s to loading sample
wash=10*60; % s to wash sample
elute=20*60; % s to elute by gradient from 0% elution buffer to 100%
total_elute=8*60; % s to elute with 100% elution buffer
Vset=0.25/60;        % mL/sec flow rate (two syringes!)
lv = 5;          % mm/mL, length per volume for syringe
rs = 150;        % degree/mm rotation in degrees per length
gdf = 24;         % -, gear-down factor, one gearbox

t0=0;
t1=load
t2=load+wash
t3=load+wash+elute
t4=load+wash+elute+total_elute
Vy=(Vset+(Vset/(t3-t2)*t2))*(tt-t2)+0.5*(Vset/(t3-t2))*(t2^2-tt^2);
% integrated V between t2&t3 for buffer 20 mM Imidazole
y = Vy*lv*rs*gdf % °, degrees of rotation motor port C
Vz=Vset*(tt-t2)-Vy
% integrated V between t2&t3 for buffer 500 mM Imidazole
z = Vz*lv*rs*gdf % °, degrees of rotation motor port D

plot(tt, y,'go')
plot(tt, z,'co')
xlabel('time, s');
ylabel('rotation, #');
legend('rotation set')
hold on
end
```

4. Save functions ‘set_rotationD.m‘ for step D as a separate MATLAB file and adjust parameter of interest:

```
function y = set_rotationD(tt)

%%% PARAMETER MUST BE ADJUSTED %%%
load=8*60; % s to loading sample
wash=10*60; % s to wash sample
elute=20*60; % s to elute by gradient from 0% elution buffer to 100%
total_elute=8*60; % s to elute with 100% elution buffer
Vset=0.25/60;        % mL/sec flow rate (two syringes!)
lv = 5;          % mm/mL, length per volume for syringe
rs = 150;        % degree/mm rotation in degrees per length
gdf = 24;         % -, gear-down factor, one gearbox

t0=0;
t1=load
t2=load+wash
t3=load+wash+elute
t4=load+wash+elute+total_elute
Vt = Vset*(tt-t3);% integrated V dosed between t3&t4
y = Vt*lv*rs*gdf % °, degrees of rotation

plot(tt, y,'bo')
xlabel('time, s');
ylabel('rotation, #');
legend('rotation set')
hold on
```

```
end
```

5. After installing the MATLAB Support Package for LEGO-MINDSTORMS EV3 Hardware the EV3 intelligent brick must be connected with the MATLAB program.

   Therefore three options are available:

```
% Wifi connection:
myev3 = legoev3('wifi','IP address,'BrickID');
% Bluetooth connection:
myev3 = legoev3(' Bluetooth','PortID');  %Port-ID must be adapted
% USB connection:
myev3 = legoev3('USB');
```

6. Ports must be matched with servo motors (in this case during 7):

```
mymotor = motor(myev3,'A'); % connection ports A,B,C or D
```

   To connect more than one motor simultaneously name motors and change this accordingly in the whole script:

```
mymotorA = motor(myev3,'A'); % connection ports A,B,C or D
mymotorB = motor(myev3,'B'); % connection ports A,B,C or D
```

7. Adjust parameters then run the program. It will start the motor, compare the actual rotation with the rotations needed and adjust them:

```
%%% PARAMETER MUST BE ADJUSTED %%%
load=8*60; % s to loading sample
wash=10*60; % s to wash sample
elute=20*60; % s to elute by gradient from 0% elution buffer to 100%
total_elute=8*60; % s to elute with 100% elution buffer

t0=0;
t1=load
t2=load+wash
t3=load+wash+elute
t4=load+wash+elute+total_elute
tic;                    %start counter

%% LOAD %%
mymotorA = motor(myev3,'A'); % connection to port A
start(mymotorA);             % start motor
resetRotation(mymotorA);     % set rotation counter to zero
mymotorA.Speed = 2;          % set initial motor speed
tt=toc;                 % read out actual time point
i=0; % counter
while i<t1
i=toc;
%%%compare slopes rotation_actual and rotation_set%%%
  tt=toc;
  rotation_act_1 = readRotation(mymotorA);
  rotation_set_1=set_rotationA(tt);
  pause(0.001);
  tt=toc;
  rotation_act_2 = readRotation(mymotorA)
  rotation_set_2=set_rotationA(tt);
  rot_set=rotation_set_2-rotation_set_1;
  rot_act=rotation_act_2-rotation_act_1;
%%% adjust motor-speed %%%
    if (rot_set<rot_act);
      if mymotorA.Speed>2;
      mymotorA.Speed = mymotorA.Speed-1;
      else mymotorA.Speed=2;
      end
    else
```

```
      if (rot_set==rot_act);
      mymotorA.Speed = mymotorA.Speed;
      else mymotorA.Speed = mymotorA.Speed+2;
      end
    end
plot(tt, rotation_act_2,'bs');
xlabel('time, s');
ylabel('rotation, #');
hold on;
q=mymotorA.Speed;
end

stop(mymotorA);
resetRotation(mymotorA); % set rotation counter to zero

%% WASH %%
mymotorB = motor(myev3,'B'); % connection to port B
start(mymotorB);           % start motor
resetRotation(mymotorB); % set rotation counter to zero
mymotorB.Speed = q;        % set initial motor speed
tt=toc;                 %read out actual time point
j=toc; % counter
while t2>j;
    j=toc;
%%%compare slopes rotation_actual and rotation_set%%%
  tt=toc;
  rotation_act_1 = readRotation(mymotorB);
  rotation_set_1=set_rotationB(tt);
  pause(0.001);
  tt=toc;
  rotation_act_2 = readRotation(mymotorB);
  rotation_set_2=set_rotationB(tt);
  rot_set=rotation_set_2-rotation_set_1;
  rot_act=rotation_act_2-rotation_act_1;
%%% adjust motor-speed %%%
    if (rot_set<rot_act);
        if mymotorB.Speed>2;
        mymotorB.Speed = mymotorB.Speed-1;
        else mymotorB.Speed=2;
        end
    else
        if (rot_set==rot_act);
        mymotorB.Speed = mymotorB.Speed;
        else mymotorB.Speed = mymotorB.Speed+2;
        end
    end
plot(tt, rotation_act_2,'rs');
xlabel('time, s');
ylabel('rotation, #');
hold on
q=mymotorB.Speed;
end

stop(mymotorB);
resetRotation(mymotorB); % set rotation counter to zero
time=toc;

%%CHECK POINT; please control, if pumps are connected%%
a = input('Are pumps connected (y/n)? ','s')
if strcmpi(a,'y');
tic % restart counter

%% ELUTE %%
mymotorC = motor(myev3,'C'); % connection to port C
mymotorD = motor(myev3,'D'); % connection to port D
```

```
start(mymotorC);            % start motor
start(mymotorD);            % start motor
resetRotation(mymotorC); % set rotation counter to zero
resetRotation(mymotorD); % set rotation counter to zero
mymotorC.Speed = q;         % set initial motor speed
mymotorD.Speed = 0;         % set initial motor speed
k=toc+time;
while t3>k;
  k=toc+time;
%%%compare slopes rotation_actual and rotation_set%%%
  tt=toc+time;
  [y,z]=set_rotationC(tt);
  rotation_act_1C = readRotation(mymotorC);
  rotation_act_1D = readRotation(mymotorD);
  rotation_set_1C=y;
  rotation_set_1D=z;
  pause(0.001);
  tt=toc+time;
  [y,z]=set_rotationC(tt);
  rotation_act_2C = readRotation(mymotorC);
  rotation_act_2D = readRotation(mymotorD);
  rotation_set_2C=y;
  rotation_set_2D=z;
  rot_setC=rotation_set_2C-rotation_set_1C;
  rot_actC=rotation_act_2C-rotation_act_1C;
  rot_setD=rotation_set_2D-rotation_set_1D;
  rot_actD=rotation_act_2D-rotation_act_1D;
%%% adjust motor-speed C%%%
  if (rot_setC<rot_actC);
        if mymotorC.Speed>2;
        mymotorC.Speed = mymotorC.Speed-1;
        else mymotorC.Speed=2;
        end
  else
        if (rot_setC==rot_actC);
        mymotorC.Speed = mymotorC.Speed;
        else mymotorC.Speed = mymotorC.Speed+2;
        end
  end
%%% adjust motor-speed D%%%
   if (rot_setD<rot_actD);
        if mymotorD.Speed>2;
        mymotorD.Speed = mymotorD.Speed-1;
        else mymotorD.Speed=1;
        end
   else
        if (rot_setD==rot_actD);
        mymotorD.Speed = mymotorD.Speed;
        else mymotorD.Speed = mymotorD.Speed+2;
        end
   end
   q=mymotorD.Speed;

plot(tt, rotation_act_2C,'gs')
plot(tt, rotation_act_2D,'cs')
xlabel('time, s');
ylabel('rotation, #');
hold on;
end

stop(mymotorC);
resetRotation(mymotorC);
resetRotation(mymotorD);% set rotation counter to zero
```

```
%% TOTAL ELUTE
mymotorD.Speed = q;        % set initial motor speed
l=toc+time;               %read out actual time point
while l<t4
l=toc+time;
%%%compare slopes rotation_actual and rotation_set%%%
  tt=toc+time;
  rotation_act_1 = readRotation(mymotorD);
  rotation_set_1=set_rotationD(tt);
  pause(0.001);
  tt=toc+time;
  rotation_act_2 = readRotation(mymotorD);
  rotation_set_2=set_rotationD(tt);
  rot_set=rotation_set_2-rotation_set_1;
  rot_act=rotation_act_2-rotation_act_1;
%%% adjust motor-speed %%%
    if (rot_set<rot_act);
        if mymotorD.Speed>2;
        mymotorD.Speed = mymotorD.Speed-1;
        else mymotorD.Speed=2;
        end
    else
        if (rot_set==rot_act);
            mymotorD.Speed = mymotorD.Speed;
        else mymotorD.Speed = mymotorD.Speed+2;
        end
        q=mymotorD.Speed;
    end
    q

plot(tt, rotation_act_2,'bs')
xlabel('time, s');
ylabel('rotation, #');
hold on
end
stop(mymotorD);
resetRotation(mymotorD); % set rotation counter to zero
end
```

8. Trouble shooting:

Depending on the correlation of the syringe type, the duration of the process, as well as the adjusted flow rate the rotation performance might reach the limits of the dosing unit. This is a result of exceeding the maximal or minimal rotation of the motor block. To avoid this, several adjustments can be done: To vary the speed of the dosing the **gear-down factor** can be changed by altering the number or type of **gearboxes**. If this is not sufficient, another **syringe** can be used to dose a higher or smaller volume per rotation. Furthermore, the size of the steps for **motor-speed adaption** can be enlarged to reach bigger changes in a shorter period of time. Keep in mind that enlarging the step size means enlarging the fluctuation. In general, we recommend adapting the syringe type. Please check parameter r_S. Aging and pressure can cause variation. If pressure destabilises the housing, additional bricks can be added as cross strives.

Performing a fed-batch process

1. Save function ‘set_rotation.m‘ as a separate MATLAB file and adjust parameter of interest:

```
function y = set_rotation (tt,mue)

%%% PARAMETERS MUST BE ADJUSTED %%%
mue = 0.5;  % 1/h, set growth rate
cs0 = 18;    % mg/ml, substrate concentration
cx0 = 0.04;  % mg/ml, starting cell concentration
lv = 8;      % mm/ml, length per volumen for syringe
rs = 90;     % degree/mm rotation in degrees per length
gdf = 576;   % -, gear-down factor (slow down)
VR= 50;      % ml, starting volume
Yxs = 0.35;  % g(x)/g(s), cell yield

y =(cx0*VR/(Yxs*cs0))*(exp(mue*(tt./3600))-1)*lv*rs*gdf;%°,rotation
end
```

2. After installing the MATLAB Support Package for LEGO-MINDSTORMS EV3 Hardware the EV3 intelligent brick must be connected with the MATLAB program.

   Therefore, three options are available:

```
% Wifi connection:
myev3 = legoev3('wifi','IP address,'BrickID');
% Bluetooth connection:
myev3 = legoev3(' Bluetooth','Port-ID'); %Port-ID must be adapted
% USB connection:
myev3 = legoev3 ('USB');
```

3. After that ports must be matched with servo motors.

```
mymotor = motor(myev3,'A'); % connection ports A,B,C or D
```

   To connect more than one motor simultaneously name motors and change it in the whole script:

```
mymotorA = motor(myev3,'A'); % connection ports A,B,C or D
mymotorB = motor(myev3,'B'); % connection ports A,B,C or D
```

4. Choose growth rate and process duration. Run the program. It will start the motor, compare the actual rotation with the rotations needed and adjust it:

```
%%% PARAMETERS MUST BE ADJUSTED %%%
mue = 0.5;               % 1/h, set growth rate
t_end = 10;              % min process time
mymotor.Speed = 5;        % initial motorspeed

%start values
resetRotation(mymotor)    % set rotation counter to zero
tic                       %start counter
tt = toc;
rotation = 0;
i = 1;
% plot
m_rot = zeros(floor(t_end*1.1),1);
plot(0:100:6000, set_rotation(0:100:6000,mue));
Fig
axis([0 (t_end+1)*60 0 set_rotation((t_end+1)*60, mue) ]);
xlim([0 (t_end+1)*60]);
ylim([0 set_rotation((t_end+1)*60,mue)]);
hold on
plot(gca,0, 0, 'ro', 0, 0, 'bs');
hold on
```

```
% process loop
while(tt < t_end*60)
    m_rot(i) = mymotor.Speed;
    i = i+1;
    start(mymotor);%Start
    pause(27);
    stop(mymotor);
    pause(3);
    rotation = readRotation(mymotor);
    tt = toc;
    plot(gca,tt, rotation, 'ro', tt, set_rotation (tt,mue), 'bs');
    hold on

%%% adjust motor-speed %%%
    if(rotation > set_rotation (tt,mue))
        if(mymotor.Speed <= 5)
             mymotor.Speed = 0;
        else mymotor.Speed = mymotor.Speed-1;
        end
    else mymotor.Speed = mymotor.Speed+1;
        if(mymotor.Speed >= 90)
           mymotor.Speed = 90;
        end
    end
end
stop(mymotor);
```
