# Supplementary material for: An automated and parallelised DIY-dosing unit for individual and complex feeding profiles: Construction, validation and applications
Source: PLoS One. 2019 Jun 19;14(6):e0217268. doi: 10.1371/journal.pone.0217268 (PMC6583958; doi:10.1371/journal.pone.0217268)
Supplement: S2 File — S2 Table depicts the calculated values for the transformation of motor speed into flow rates. S2 Fig shows (A) a picture of one dosing unit connected to the EV3 brick and (B) the resulting flow rates for 20 mL syringe for different motor speeds. (PDF) [file pone.0217268.s004.pdf]

Supporting Information 3

Calculated values

		motor speed, %	0	5	10	20	30	40	50	60	70	80	90	100
°rot per 10 s	° $(10\ s)^{-1}$	I	0	558.954	1028	1959	2936	3861	4780	5678	6564	7503	8389	9443
		II	0	558.954	1060	1978	2946	3854	4781	5676	6578	7531	8405	9476
		III	0	558.954	1071	1980	2944	3861	4783	5684	6570	7521	8398	9479
		average	0	558.954	1053	1972.333	2942	3858.667	4781.333	5679.333	6570.667	7518.333	8397.333	9466
		standard deviation	0	0	22.33831	11.59023	5.291503	4.041452	1.527525	4.163332	7.023769	14.1892	8.020806	19.97498
		rotation per second	0	55.8954	105.3	197.2333	294.2	385.8667	478.1333	567.9333	657.0667	751.8333	839.7333	946.6
1mL syringe	mL min^{-1}	0 gearbox	0	0.392248	0.738947	1.384094	2.064561	2.707836	3.355322	3.985497	4.610994	5.276023	5.892865	6.642807
		1 gearbox	0	0.016344	0.030789	0.057671	0.086023	0.112827	0.139805	0.166062	0.192125	0.219834	0.245536	0.276784
		2 gearbox	0	0.000681	0.001252	0.002387	0.003577	0.004704	0.005824	0.006918	0.007997	0.009141	0.010221	0.011505
10mL syringe		0 gearbox	0	2.79477	5.265	9.861667	14.71	19.29333	23.90667	28.39667	32.85333	37.59167	41.98667	47.33
		1 gearbox	0	0.116449	0.219375	0.410903	0.612917	0.803889	0.996111	1.183194	1.368889	1.566319	1.749444	1.972083
		2 gearbox	0	0.004852	0.009141	0.017121	0.025538	0.033495	0.041505	0.0493	0.057037	0.065263	0.072894	0.08217
20mL syringe		0 gearbox	0	4.471632	8.424	15.77867	23.536	30.86933	38.25067	45.43467	52.56533	60.14667	67.17867	75.728
		1 gearbox	0	0.186318	0.351	0.657444	0.980667	1.286222	1.593778	1.893111	2.190222	2.506111	2.799111	3.155333
		2 gearbox	0	0.007763	0.014625	0.027394	0.040861	0.053593	0.066407	0.07888	0.091259	0.104421	0.11663	0.131472
50mL syringe		0 gearbox	0	12.4212	23.4	43.82963	65.37778	85.74815	106.2519	126.2074	146.0148	167.0741	186.6074	210.3556
		1 gearbox	0	0.51755	0.975	1.826235	2.724074	3.57284	4.42716	5.258642	6.083951	6.96142	7.775309	8.764815
		2 gearbox	0	0.021565	0.040625	0.076093	0.113503	0.148868	0.184465	0.21911	0.253498	0.290059	0.323971	0.365201

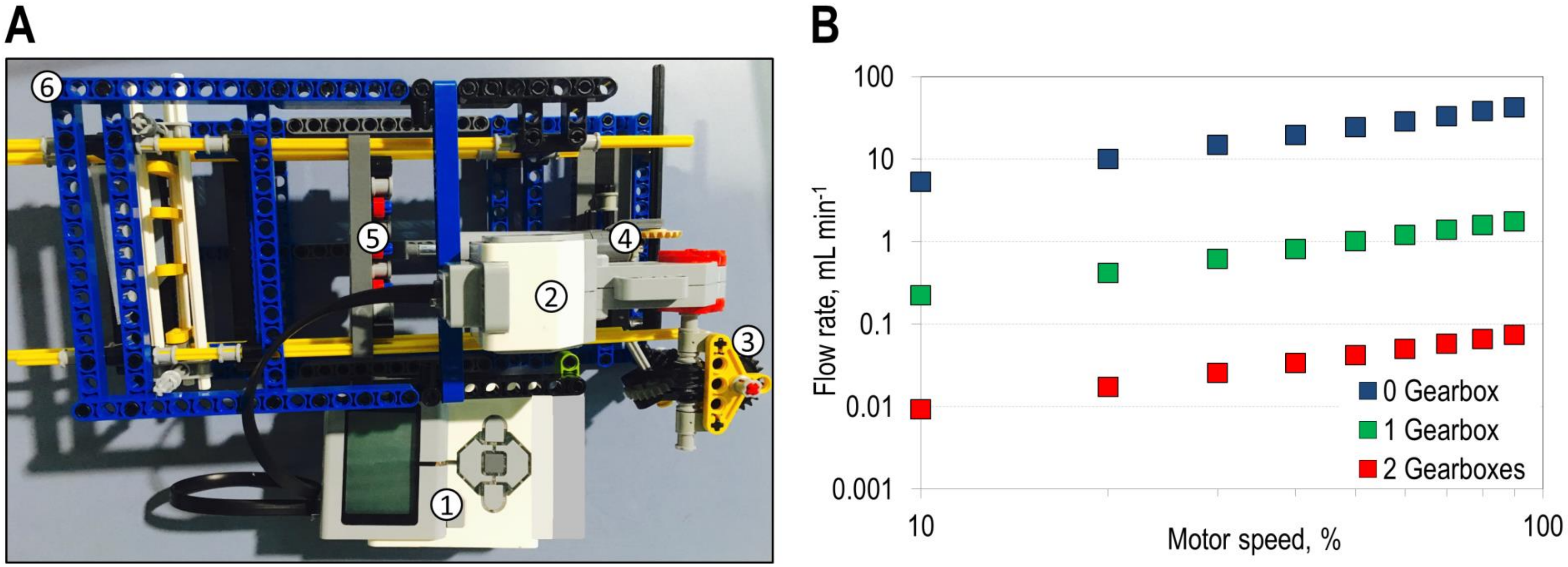

Fig S3: Picture of one dosing unit (A) connected to the programmable MINDSTORMS-EV3 brick (A.1) and the resulting flow rates for 20 mL syringes at different motor speeds as an example (B). The dosing unit consists of a EV3 large servo motor (A.2), gear-down modules (the prototype version contains two gearboxes) (A.3), a linear actuator (A.4), the syringe clamping device (A.5) and the housing (A.6) which is stabilising the construct. Depending on the syringe type, the number of gearboxes, and the motorspeed different flow rates can be performed (B). For 20 mL syringes flow rates can range from around 0.01 to 0.13 mL h^{-1} with 2 gearboxes (red), over 0.19 to 3.16 mL h^{-1} (1gearbox, green) up to 4.47 to75.73 mL h^{-1} with no gearboxes (blue).
