# Supplementary material for: An automated and parallelised DIY-dosing unit for individual and complex feeding profiles: Construction, validation and applications
Source: PLoS One. 2019 Jun 19;14(6):e0217268. doi: 10.1371/journal.pone.0217268 (PMC6583958; doi:10.1371/journal.pone.0217268)
Supplement: S3 File — S3 Fig (A) Define functions of the dosing profile, (B) Calculated formulas, (C) Calculation of complex dosing profiles and (D) the actual sum of rotations. (PDF) [file pone.0217268.s005.pdf]

Supporting Information 3
General derivation of dosing functions:

Functions for flow rate over time

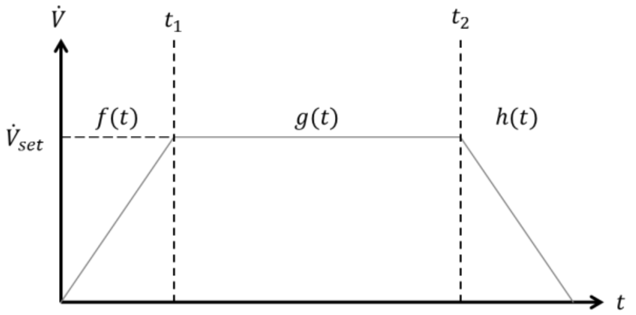

$$f(t) = \frac{\dot{V}_{set}}{t_1 - t_0} * t$$

$$g(t) = \dot{V}_{set}$$

$$h(t) = \dot{V}_{set} - \frac{\dot{V}_{set}}{t_3 - t_2} * (t - t_2)$$

Fig S3.A: Define fuctions of the dosing profile.

Formulas for individual integrals:

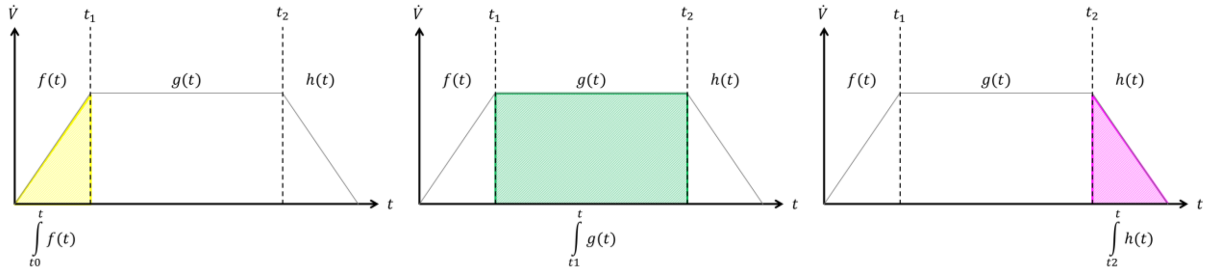

Fig S3.B: Calculate formulas for each individual fuction

$$\int_{t0}^{t} f(t)dt = \left|\frac{1}{2} * \frac{\dot{V}_{set}}{t_1 - t_0} * t^2\right|_{t0}^{t} = \frac{1}{2} * \frac{\dot{V}_{set}}{t_1 - t_0} * t^2$$

$$\int_{t1}^{t} g(t)dt = \left|\dot{V}_{set} * t\right|_{t1}^{t} = \dot{V}_{set} * t - \dot{V}_{set} * t_1$$

$$\int_{t2}^{t} h(t)dt = \left|\dot{V}_{set} * t - \frac{1}{2} * \frac{\dot{V}_{set}}{t_3 - t_2} * t^2 + \frac{\dot{V}_{set}}{t_3 - t_2} * t_2 * t\right|_{t2}^{t}$$

$$\int_{t2}^{t} h(t)dt = (\dot{V}_{set} + \frac{\dot{V}_{set}}{t_3 - t_2} * t_2) * t - \frac{1}{2} * \frac{\dot{V}_{set}}{t_3 - t_2} * t^2 - ((\dot{V}_{set} + \frac{\dot{V}_{set}}{t_3 - t_2} * t_2) * t_2 - \frac{1}{2} * \frac{\dot{V}_{set}}{t_3 - t_2} * {t_2}^2)$$

Integration of overall process needs the combination of individual integrals:

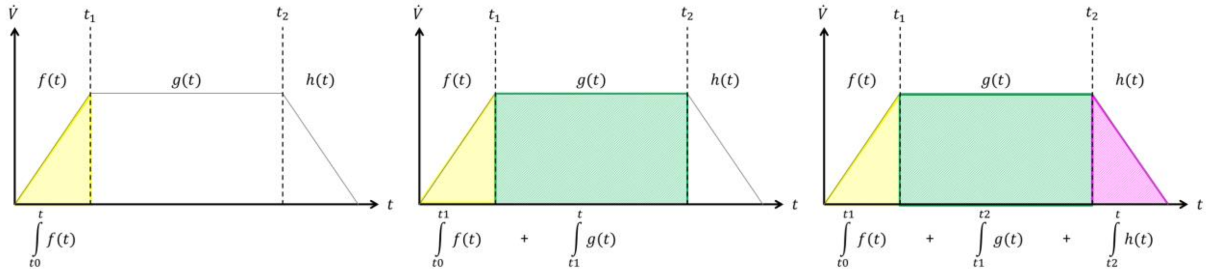

Fig S3.C: Calculation of a complex dosing profile consisting of different phases. Heterologous changes in flow rate are described in several phases (I,II, and III). For each section an time (t) dependent equation is defined (f(t),g(t), and h(t)). The dosed volume at time point t can be expressed as the appropriate integral of the functions. The volume of already completed phases must be summed up with the current transitory stage of the actual phase.

For dosing in Phase I:

$$V(t) = \frac{1}{2} * \frac{\dot{V}_{set}}{t_1 - t_0} * t^2$$

For dosing in Phase II:

$$V(t) = \frac{1}{2} * \frac{\dot{V}_{set}}{t_1 - t_0} * {t_1}^2 + \dot{V}_{set} * t - \dot{V}_{set} * t_1$$

For dosing in Phase III:

$$V(t) = \frac{1}{2} * \frac{\dot{V}_{set}}{t_1 - t_0} * {t_1}^2 + \dot{V}_{set} * t_2 - \dot{V}_{set} * t_1 + (\dot{V}_{set} + \frac{\dot{V}_{set}}{t_3 - t_2} * t_2) * (t - t_2) + \frac{1}{2} * \frac{\dot{V}_{set}}{t_3 - t_2} * ({t_2}^2 - t^2)$$

'Sum of rotations' diagram for a 30 second example of here presented, representative profile:

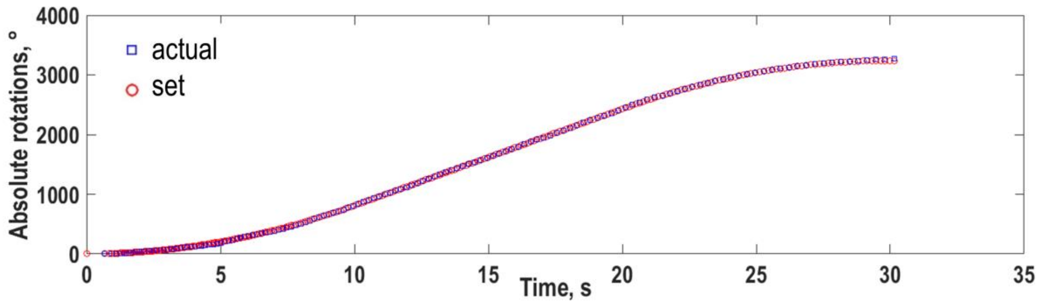

Fig S3.D: The actual sum of rotation is oscillating around the set number of rotations. To accomplish adequate small steps and guarantee a continual flow the actual sum of rotations is oscillating around the set number of rotation. As shown in the zooms, the fluctuations of the exponential feeding profile are in the scale of a few seconds.
