# Supplementary material for: An automated and parallelised DIY-dosing unit for individual and complex feeding profiles: Construction, validation and applications
Source: PLoS One. 2019 Jun 19;14(6):e0217268. doi: 10.1371/journal.pone.0217268 (PMC6583958; doi:10.1371/journal.pone.0217268)
Supplement: S4 File — Parameters and Equations for the derivation, the figure shows the C-source consumption over cell dry weight production. (PDF) [file pone.0217268.s006.pdf]

Supporting Information 5
Derivation of dosing function for fed-batch processes

Parameters:

$$t = time\ point\ of\ interest, \text{ h}$$
$$V_R = volume\ of\ reactor\ at\ time\ point\ t, \text{ mL}$$
$$\dot{V}_{in} = volumetric\ flow\ rate;\ influx, \text{ mL h}^{1}$$

$$c_s = substrate\ concentration\ in\ the\ reaction\ vessel\ at\ time\ point\ t, \text{ mg mL}^{-1}$$
$$c_{s0} = substrate\ concentration\ in\ the\ feed, \text{ mg mL}^{-1}$$
$$m_S = mass\ of\ substrate\ in\ the\ reaction\ vessel\ at\ time\ point\ t, \text{ mg}$$
$$q_S = substrate\ uptake\ rate, \text{ mg}_{\text{Substrat}}\text{ mg}_{\text{CDW}}^{-1}\text{ h}^{-1}$$
$$Y_{XS} = biomass\ yield, \text{ mg}_{\text{CDW}}\text{ mg}_{\text{Substrate}}^{-1}$$
$$\mu = growth\ rate, \text{ h}^{-1}$$
$$c_X = \ biomass\ concentration\ in\ the\ reaction\ vessel\ at\ time\ point\ t, \text{ mg}_{\text{CDW}}\text{ mL}^{-1}$$
$$c_{X0} = \ biomass\ concentration\ at\ starting\ point\ t, \text{ mg}_{\text{CDW}}\text{ mL}^{-1}$$

$$V_F(t) = volume\ of\ dosed\ feed\ at\ time\ point\ t, \text{ mL}$$
$$gdf = gear\ down\ factor, \text{ -}$$
$$l_V = length\ per\ volumen\ for\ syringe, \text{ mm mL}^{-1}$$
$$r_S = degree/mm\ rotation\ in\ degrees\ per\ length, \text{ ° mm}$$
$$set\_rotation(t) = degrees\ of\ rotation\ at\ t, \text{ °}$$

Equations:

$$m_S = V_R * c_S$$

$$q_S = \frac{\mu}{Y_{XS}}$$

$$c_x = c_{x0} * e^{\mu * t}$$

$$set\_rotation(t) = V_F(t) * l_V * r_S * gdf$$

Assumption: $dm_S \xrightarrow{limited} 0$, constant reactor volume

$$\frac{d(V_R * c_S)}{dt} = (\dot{V}_{in} * c_{S0} - c_x \cdot q_S * V_R)$$

$$\frac{d(m_S)}{dt} = (\dot{V}_{in} * c_{S0} - c_x \cdot q_S * V_R)$$

$$0 = (\dot{V}_{in} * c_{S0} - c_x \cdot q_S * V_R)$$

$$\dot{V}_{in} = \frac{c_x \cdot q_S * V_R}{c_{S0}} = \frac{c_x \cdot \mu * V_R}{Y_{XS} * c_{S0}}$$

$\frac{dc_x}{dt}$ is time dependent => $c_x = c_{x0} * e^{\mu * t}$;

$$V_F(t) = \int_0^t \dot{V}_{in}(t)\,dt = \left| \frac{1}{\mu} * \frac{\mu * V_R * c_{X0}}{Y_{XS} * c_{S0}} * e^{\mu * t} \right|_0^t$$

$V_F(t) = \frac{V_R * c_{X0}}{Y_{XS} * c_{S0}} * (e^{\mu * t} - 1)$Total volume dosed can be transformed into the dosing function for total rotation (in the script time is tracked in seconds! Please adjust either µ or t):

$$set\_rotation(t) = \frac{V_R * c_{X0}}{Y_{XS} * c_{S0}} * (e^{\mu * t} - 1) * gdf * l_v * r_S$$

Determination of biomass yield:

Y_{XS} was determined for Wildtype strain HMS174(DE3) by a batch process under the same conditions with 20 mM Glucose as C-source.

Time, h	CDW, mg mL^{-1}	C-source, mg mL^{-1}
0	0.046666	2.187584
1.5	0.101678	2.082808
3	0.212959	1.752081
4.5	0.418738	1.013999
6	0.819527	0

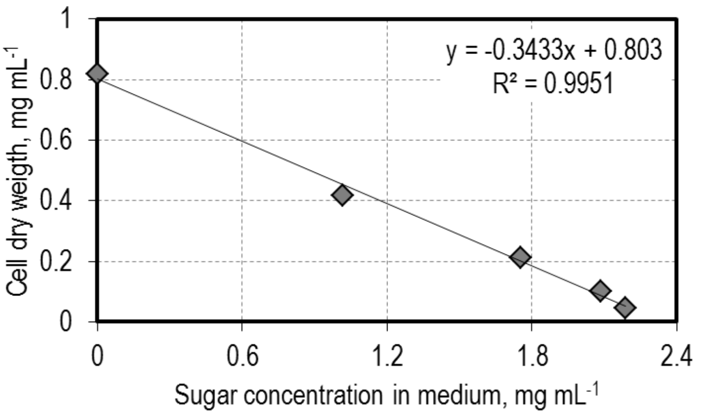

Fig S5: C-source consumption was monitored over cell dry weight production. The resulting ratio is known as biomass yield Y_{XS}.
