# Supplementary material for: An automated and parallelised DIY-dosing unit for individual and complex feeding profiles: Construction, validation and applications
Source: PLoS One. 2019 Jun 19;14(6):e0217268. doi: 10.1371/journal.pone.0217268 (PMC6583958; doi:10.1371/journal.pone.0217268)
Supplement: S5 File — The distribution of cellular resources provided by substrate uptake is shown. (PDF) [file pone.0217268.s007.pdf]

Supporting Information 6
Calculation of the energy demand of heterologous load

The differences in biomass yields are constituted in the need of resources, which are recruited for the additional tasks performed in producing cells. Calculations are simplified by the assumption that $q_{S,maintenance} << q_{S,\mu}$ and can therefore be neglected. Side product formation can also be neglected, because in C-source limited processes side product formation is extremely reduced as described in the chapter **'Setting growth rates in parallel shaking flasks'**.

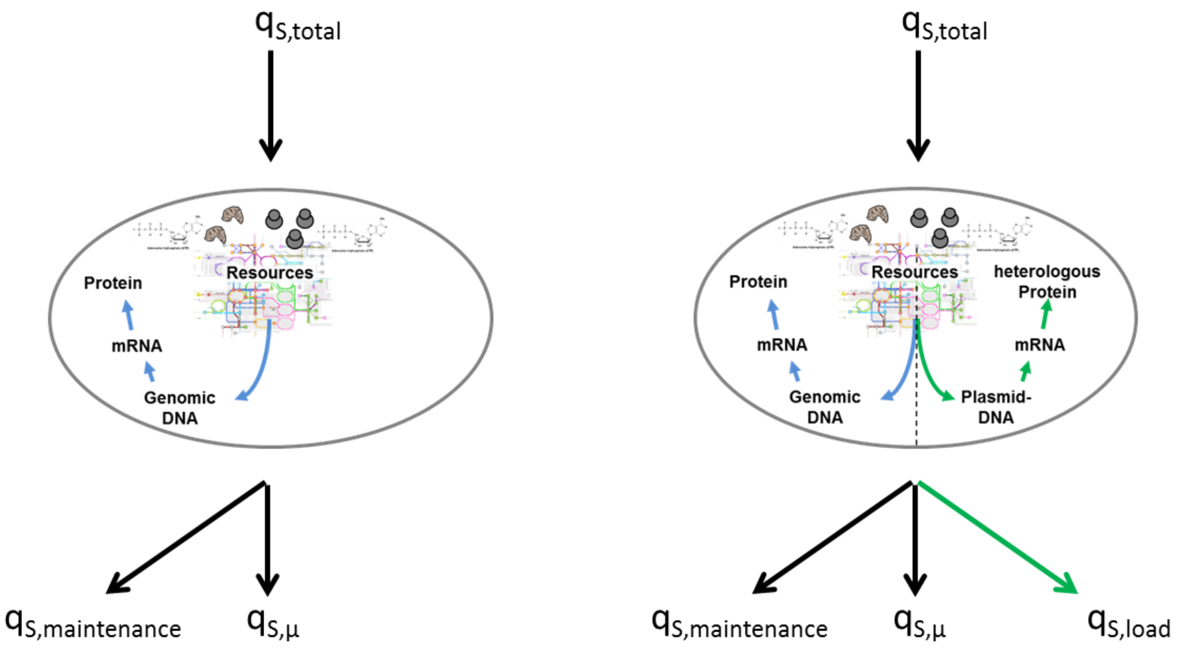

Fig S6: Distribution of cellular resources provided by substrate uptake. In wild type cells substrate uptake was used to perform growth and maintenance exclusively (left). The production of heterologous loads consumes a significant part of the substrate taken up (right).

<u>Parameters</u>

- $q_{S,total} = total\ substrate\ uptake\ needed\ for\ growth$, $mg_{Substrate}\ mg_{CDW}^{-1}\ h^{-1}$

- $\mu_{WT} = growth\ rate\ of\ the\ wild\ type$, h^{-1}
  $Y_{XS,WT} = biomass\ yield\ of\ the\ wild\ type$, $mg_{CDW}\ mg_{Substrate}^{-1}$
  $q_{S,\mu} = growth\ specific\ substrate\ demand$, $mg_{Substrate}\ mg_{CDW}^{-1}\ h^{-1}$

- $\mu_{loaded} = growth\ rate\ of\ the\ loaded\ strain$, h^{-1}
  $Y_{XS,loaded} = biomass\ yield\ of\ the\ loaded\ strain$, $mg_{CDW}\ mg_{Substrate}^{-1}$
  $q_{S,load} = load\ specific\ substrate\ demand$, $mg_{Substrate}\ mg_{CDW}^{-1}\ h^{-1}$

<u>Derivation</u>

In general growth is determined by a constant biomass yield and a specific substrate uptake rate:

$$\mu = Y_{XS} * q_{S,total}$$

In case of a loaded strain the replication of the plasmid forms part of the cellular growth. To this end, the total substrate uptake contains a load-specific demand $q_{S,load}$ in addition to the growth specific substrate demand $q_{S,\mu}$. Based on the data shown in Fig 8.B, the assumption was made that plasmid caused energy demand is linearly correlated with growth.

$$q_{S,total} = q_{S,\mu}(+q_{S,load})$$

For wild type strains (I) the total substrate uptake needed for growth can be described as growth specific substrate demand. To determine growth in loaded strains (II), this load specific substrate uptake is considered additionally.

(I) $\mu_{WT} = Y_{XS,WT} * q_{S,\mu}$

(II) $\mu_{loaded} = Y_{XS,loaded} * (q_{S,\mu} + q_{S,load})$

If strains are examined at similar growth rates $\mu_{WT} = \mu_{loaded}$ the equation for the load specific substrate demand $q_{S,load}$ can be solved based on the determined biomass yields.

$$Y_{XS,WT} * q_{S,\mu} = Y_{XS,loaded} * q_{S,\mu} + Y_{XS,loaded} * q_{S,load}$$

$$q_{S,load} = q_{S,\mu} * \frac{Y_{XS,WT} - Y_{XS,loaded}}{Y_{XS,loaded}}$$

By adding the slopes, measured in Fig 8.B an energy demand $q_{S,load}$ for the plasmid of 32.6 % $q_{S,\mu}$ was calculated.

$$q_{S,load} = q_{S,\mu} * \frac{0.3555 - 0.2681}{0.2681} = q_{S,\mu} * 0.326$$
