# Supplementary material for: An automated and parallelised DIY-dosing unit for individual and complex feeding profiles: Construction, validation and applications
Source: PLoS One. 2019 Jun 19;14(6):e0217268. doi: 10.1371/journal.pone.0217268 (PMC6583958; doi:10.1371/journal.pone.0217268)
Supplement: S7 File — (PDF) [file pone.0217268.s009.pdf]

Supporting Information 9

Unit	Meaning
€	Euro
USD	US dollar
µL	Mikroliter
mL	Milliliter
L	Liter
ms	Milliseconds
min	Minutes
h	Hours
%	Percentage
nm	Nanometer
mm	Millimeter
cm	Centimeter
dm^3	Cubic decimeters
MHz	Megahertz
MB	Megabyte
GB	Gigabyte
N cm	Newton centimeter
rpm	Rounds per minute
bar	Bar
°	Degrees of rotation
°C	Degrees Celcius
mM	Millimolar
kg	Kilogram
g	Gram
$mg_{Substrate}$	Milligram of substrate
mg_{CDW}, g_{CDW}	Milligram or gram of cell dry weigth (CDW)
$mmol_{xxx}$	Millimol of molecule xxx

Abbreviations	Meaning
E. coli	*Escherichia coli* HMS174(DE3)
P. putida	*Pseudomonas putida KT2440 EM178*
DIY	Do-it-yourself
HPLC	High-pressure liquid chromatography
LIS	Liquid Injection System
RI	Refractive index detector
IMAC	Immobilised metal-affinity chromatography
CV	Column volume
EDTA	Ethylenediaminetetracetic acid
eGFP	Enhanced green fluorescent protein
RAM	Random-Access Memory
SDHC	Secure Digital High Capacity
WLAN	Wireless Local Area Network
USB	Universal Serial Bus
LB	Lysogeny broth
C-source	Carbon source
GE	General Electric
NGAM	Non-growth associated maintenance

Variables	Meaning	Unit
$\dot{V}_{in}$	Influx/ volume dosed	mL h^{-1}
$\dot{V}_{set}$	Flow rate set	mL h^{-1}
$\dot{V}_{in}(t)$	Flow rate of substrate needed for growth rate set at t	mL h^{-1}
$\dot{V}_{max}$	Maximal flow rate at 100 % motor speed	mL min^{-1}
$\dot{V}_{min}$	Minimal flow rate at 5 % motor speed	mL min^{-1}
µ	Growth rate	h^{-1}
μ_{actual}	Growth rate actual	h^{-1}
μ_{set}	Growth rate set	h^{-1}
$V_F(t)$	Total volume dosed at t	mL
V_R	Volume of reaction vessel	mL
$V_{total,max}$	Maximal total volume per syringe	mL
$Y_{XS,WT}$	Biomass yield in wild type strain	mg_{CDW} $mg_{Substrate}^{-1}$
$Y_{XS,loaded}$	Biomass yield in strain with heterologous load	mg_{CDW} $mg_{Substrate}^{-1}$
Y_{XS}	Biomass yield	mg_{CDW} $mg_{Substrate}^{-1}$
c_S	Substrate concentration in the reaction vessel	mg mL^{-1}
c_{S0}	Substrate concentration in the feed	mg mL^{-1}
c_X	Biomass concentration	mg_{CDW} mL^{-1}
c_{X0}	Initial biomass concentration	mg_{CDW} mL^{-1}
l_V	Distance per volume (syringe dependent)	mm mL^{-1}
r_S	Motor revolution per distance	° mm^{-1}
gdf	Gear-down factor	-
$set\_rotation(t)$	Degree of rotation which has to be executed at t	°
$q_{S,\mu}$	Substrate uptake rate for growth related processes	$mg_{Substrate}$ mg_{CDW}^{-1} h^{-1}
$q_{S,load}$	Substrate uptake rate depending on heterologous load	$mg_{Substrate}$ mg_{CDW}^{-1} h^{-1}
$q_{S,max}$	Maximal substrate uptake rate	$mg_{Substrate}$ mg_{CDW}^{-1} h^{-1}
$q_{S,total}$	Total substrate uptake rate	$mg_{Substrate}$ mg_{CDW}^{-1} h^{-1}
q_S	Substrate uptake rate	$mg_{Substrate}$ mg_{CDW}^{-1} h^{-1}
t	Time	s, min, h
$t_0, t_1, t_2, \ldots t_n$	Different time points 1,2,…,n	s, min, h
a	Increase in dosing rate over time	h^{-1}
b	Set point of initial volume	mL
$f(t), h(t), g(t)$	Chronological sequence of flow rates	mL h^{-1}
